# C19-Norditerpenoid Alkaloids from *Aconitum szechenyianum* and Their Effects on LPS-Activated NO Production

**DOI:** 10.3390/molecules21091175

**Published:** 2016-09-03

**Authors:** Fei Wang, Zhenggang Yue, Pei Xie, Li Zhang, Zhen Li, Bei Song, Zhishu Tang, Xiaomei Song

**Affiliations:** Shaanxi Collaborative Innovation Center of Chinese Medicinal Resource Industrialization, Shaanxi Province Key Laboratory of New Drugs and Chinese Medicine Foundation Research, Shaanxi Rheumatism and Tumor Center of TCM Engineering Technology Research, School of Pharmacy, Shaanxi University of Chinese Medicine, Xianyang 712046, China; wf88-88@163.com (F.W.); sntcm_xiepei@sina.com (P.X.); zhangl123666@163.com (L.Z.); lizhen_sntcm@sina.com (Z.L.); songbei2016@yeah.net (B.S.); tzs6565@163.com (Z.T.)

**Keywords:** *Aconitum szechenyianum*, C_19_-norditerpenoid alkaloids, anti-inflammatory activity, NO production, structure-activity relationship

## Abstract

Three new C_19_-norditerpenoid alkaloids (**1**–**3**), along with two known C_19_-norditerpenoid alkaloids (**4**–**5**) have been isolated from *Aconitum szechenyianum*. Their structures were established by extensive spectroscopic techniques and chemical methods as *szechenyianine* A (**1**), *szechenyianine* B (**2**), *szechenyianine* C (**3**), *N*-deethyl-3-acetylaconitine (**4**), and *N*-deethyldeoxyaconitine (**5**). Additionally, compounds **1**–**5** were tested for the inhibition of NO production on LPS-activated RAW264.7 cells with IC_50_ values of 36.62 ± 6.86, 3.30 ± 0.11, 7.46 ± 0.89, 8.09 ± 1.31, and 11.73 ± 1.94 μM, respectively, while the positive control drug dexamethasone showed inhibitory activity with IC_50_ value of 8.32 ± 1.45 μM. The structure-activity relationship of aconitine alkaloids were discussed.

## 1. Introduction

**Figure 1:**
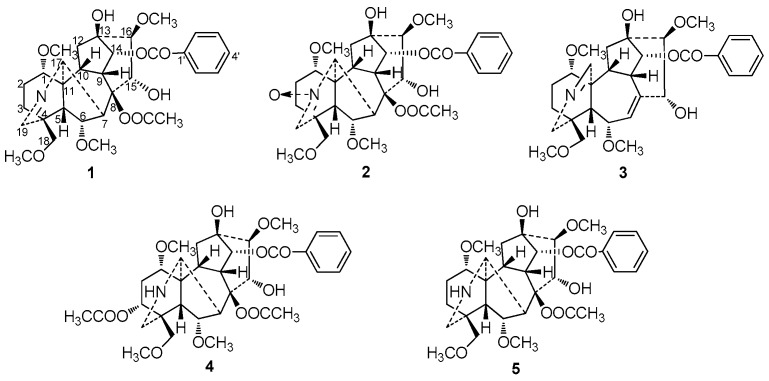
Structures of compounds **1**–**5**.

The plant *Aconitum szechenyianum* Gay., a species in the *Aconitum* genus of Ranunculaceae, is widely distributed in the west of China and used as a folk medicine in Shaanxi province, known as “Tie-Bang-Chui” [1]. Phytochemical studies revealed that *A. szechenyianum* contained mainly C_19_ and C_20_ diterpenoid alkaloids [2,3,4,5], possessing aconitine-type, 7,17-secoaconitine-type, and napeline-type skeletons. Aconitine-type have no oxygen-containing functionality at C-7, and secoaconitine-type skeleton contains N, C-17, and C-7, C-8 double bonds. Pharmacological studies revealed that these C_19_ and C_20_ diterpenoid alkaloids had demonstrated various activities as anti-inflammatory, analgesic, anticancer, anti-epileptiform, antiparasite, and cardiovascular action [6,7]. As part of our research project to explore more bioactive lead compounds from the medicinal herbs in the Qinba mountains of China [8,9,10,11,12,13,14,15,16], the chemical constituents and pharmacological studies of *A. szechenyianum* were studied, and three new C_19_-norditerpenoid alkaloids, *szechenyianine* A (**1**), *szechenyianine* B (**2**), and *szechenyianine* C (**3**), along with two known ones, *N*-deethyl-3-acetylaconitine (**4**) and *N*-deethyldeoxyaconitine (**5**) were isolated (Figure 1). Since the roots of *A. szechenyianum* were commonly used to treat rheumatism and fracture [17], the isolated compounds were evaluated for their effects on the inhibition of NO production on LPS-activated RAW264.7 cells (Table 2 and Figure 5), and the structure-activity relationship of these compounds were discussed.

## 2. Results and Discussion

**Figure 2:**
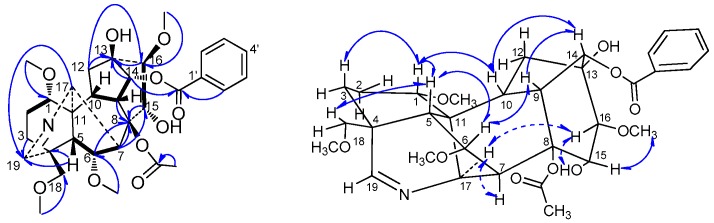
Key ^1^H-^1^H COSY (H
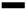
H), HMBC (H→C) and ROESY (H↔H) correlations of compound **1**.

*Szechenyianine* A (**1**) was isolated as a white amorphous powder and showed a positive reaction with Dragendorff′s reagent. Its molecular formula C_32_H_41_NO_10_ was derived from a protonated molecular ion peak at *m/z* 600.2842 [M + H]^+^ (calcd. 600.2809) of the HR-ESI-MS spectrum. Comparison of the NMR data of **1** and **5**, indicated almost similar NMR spectroscopic features, except for the number of C-4, C-17, C-19, this deduction was also confirmed by the chemical shift (Table 1) of C-4 (δ_C_ 39.0), C-19 (δ_C_ 49.0), and C-17 (δ_C_ 56.7) to upfield in ^13^C-NMR spectra of **5** compared with C-4 (δ_C_ 46.8), C-19 (δ_C_ 165.9) and C-17 (δ_C_ 60.6) of **1,** we predicted the existence of *N*=CH group in compound **1**. The ^1^H-NMR spectrum (Table 1) of **1** showed the presence of five aromatic proton signals due to a monosubstituted benzene at δ_H_ 8.02 (2H, d, *J* = 7.6 Hz), 7.55 (1H, t, *J* = 7.6 Hz), and 7.43 (2H, t, *J* = 7.6 Hz); a methine proton of an *N* = CH group at δ_H_ 7.31 (1H, s), four OMe protons at δ_H_ 3.75 (3H, s), 3.29 (3H, s), 3.18 (3H, s), and 3.03 (3H, s); and a strongly shielded proton of an acetoxyl group at δ_H_ 1.32 (3H, s). The ^13^C-NMR spectrum (Table 1) displayed 32 carbon resonances. Among them, resonances at δ_C_ 166.2, 133.6, 130.0, 129.8 (C × 2), and 128.9 (C × 2) were attributed to a benzoyloxy group; δ_C_ 61.3, 59.3, 57.4 and 56.3 were attributed to four OMe groups, δ_C_ 172.6 and 21.5 were attributed to an acetoxyl group, and the NMR features of the remained 19 resonances were characteristic to an aconitine-type alkaloid, in which δ_C_ 165.9 was attributed to a *N*=CH group and δ_C_ 74.3 and 78.9 were attributed to two oxygenated carbons associated with hydroxyl groups. The assignments of the NMR signals associated with **1** were derived from HSQC, HMBC, and ROESY experiments. In the HMBC spectrum (Figure 2), correlations of H-5 (δ_H_ 2.23) and H-17 (δ_H_ 3.97) to C-19 (δ_C_ 165.9) suggested that C-19 was involved in the *N*=CH group; correlation of H-14 (δ_H_ 4.90) to the carbonyl carbon signal of benzoyl group (δ_C_ 166.2) suggested that the benzoyl group was located at C-14; correlation of the proton signal of the acetoxyl group (δ_H_ 1.32) to C-8 (δ_C_ 90.6) suggested the acetoxyl group was located at C-8; correlations of OCH_3_ (δ_H_ 3.18) to C-1 (δ_C_ 82.3), OCH_3_ (δ_H_ 3.03) to C-6 (δ_C_ 84.1), OCH_3_ (δ_H_ 3.75) to C-16 (δ_C_ 89.9), and OCH_3_ (δ_H_ 3.29) to C-18 (δ_C_ 78.2) suggested four methoxyl groups were linked at C-1, C-6, C-16, and C-18, respectively; correlations of H-12 (δ_H_ 2.20, 2.21), H-14 (δ_H_ 4.90), and H-16 (δ_H_ 3.42) to C-13 (δ_C_ 74.3), H-9 (δ_H_ 2.70) and H-16 (δ_H_ 3.42) to C-15 (δ_C_ 78.9), suggested two hydroxyl groups were linked at C-13 and C-15, respectively. Thus, the planar structure of **1** was deduced as 14-benzoyloxy-8-acetoxyl-13,15-dihydroxy-1,6,16,18-tetramethoxy-19-en-aconitane. Meanwhile, in the ROSEY spectrum (Figure 2) of **1**, the NOE correlations of H-1/H-10, H-10/H-14, H-14/H-9, and H-9/H-6 indicated β-orientation of H-1, H-6, H-9, H-10, and H-14, and α-axial configurations of 1-OCH_3_, 6-OCH_3_ and 14-benzoyloxy; NOE correlations of H-6/H-5 and H-5/H-18 revealed β-orientation of H-18 and 18-OCH_3_, and α-axial of H-19; NOE correlations of H-17/H-7, H-16 and 15-OH, revealed α-axial of H-16, H-17 and 15-OH, and β-orientation of 16-OCH_3_, 13-OH and 8-acetoxyl. Moreover, the NOE correlations of H-1/H-3 and H-5 while no correlation between H-2 and H-5 indicated **1** had ring A (C-1, C-2, C-3, C-4, C-5, and C-11) in the chair conformation. Thus, according to the literature [18], compound **1** was assigned the name as (A-*c*)-14α-benzoyloxy-8β-acetoxyl-13β,15α-dihydroxy-1α,6α,16β,18β-tetramethoxy-19-en-aconitane.

**Figure 3:**
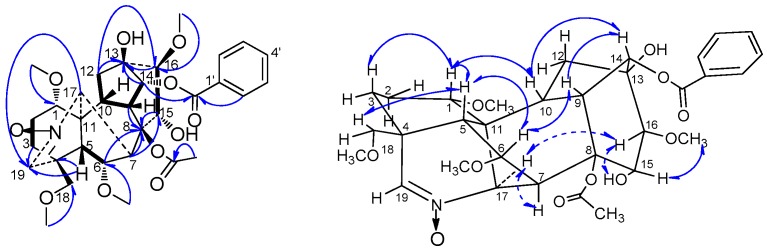
Key ^1^H-^1^H COSY (H
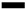
H), HMBC (H→C) and ROESY (H↔H) correlations of compound **2**.

S*zechenyianine* B (**2**) was isolated as a white amorphous powder. The NMR spectroscopic data indicated that **2** was an analogu of **1** with similar skeleton and substituent groups. However, the molecular formula of **2** was deduced as C_32_H_41_NO_11_ from the protonated molecular ion peak at *m/z* 616.2783 [M + H]^+^ (calcd. 616.2758), suggesting that an *N*-oxidation group was included in compound **2**. This deduction was also confirmed by the chemical shift (Table 1) of C-4 (δ_C_ 42.1) and C-19 (δ_C_ 138.9) to upfield, and C-17 (δ_C_ 72.9) to downfield in ^13^C-NMR spectra of **2** compared with C-4 (δ_C_ 46.8), C-19 (δ_C_ 165.9) and C-17 (δ_C_ 60.6) of **1**. Thus, compound **2** was identified by HSQC, ^1^H-^1^H COSY, HMBC, and ROESY experiments (Table 1 and Figure 3) as (A-*c*)-14α-benzoyloxy-8β-acetoxyl-13β,15α-dihydroxy-1α,6α,16β,18β-tetra-methoxy-19-en-aconitane-*N*-oxide.

**Figure 4:**
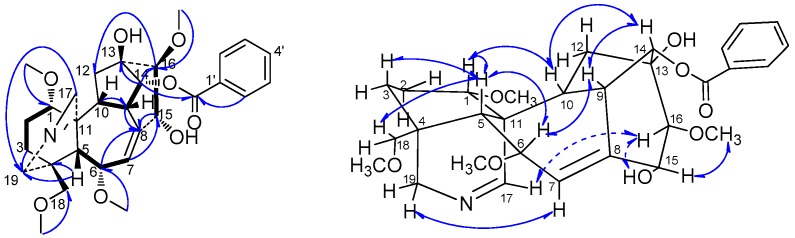
Key ^1^H-^1^H COSY (H
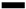
H), HMBC (H→C) and ROESY (H↔H) correlations of compound **3**.

*Szechenyianine* C (**3**) was isolated as a white amorphous powder. Its molecular formula C_30_H_39_NO_8_ was derived from a protonated molecular ion peak at *m/z* 542.2783 [M + H]^+^ (calcd. 542.2754) of the HR-ESI-MS spectrum. The ^1^H-NMR spectrum (Table 1) of **3** showed the presence of five aromatic protons signals due to a monosubstituted benzene at δ_H_ 8.03 (2H, d, *J* = 7.5 Hz), 7.53 (1H, t, *J* = 7.5 Hz), and 7.42 (2H, t, *J* = 7.5 Hz); two olefinic protons signals at δ_H_ 7.86 (1H, brs) due to *N*=CH and δ_H_ 5.62 (1H, d, *J* = 5.5 Hz) due to C=CH, respectively; and four OMe protons at δ_H_ 3.75 (3H, s), 3.27 (3H, s), 3.20 (3H, s) and 3.19 (3H, s). The ^13^C-NMR spectrum (Table 1) displayed 30 carbon resonances. Among them, resonances at δ_C_ 166.4, 133.5, 130.0, 130.1 (C × 2) and 128.7 (C × 2) were attributed to a benzoyl group; δ_C_ 61.8, 59.1, 58.2 and 56.9 were attributed to four OMe groups; and the NMR features of the remained 19 resonances were characteristic to a 7, 17-secoaconitine alkaloid, in which δ_C_ 166.4 was attributed to a *N*=CH group, and δ_C_ 132.1 and 137.5 were attributed to an olefinic bond. In the HMBC spectrum (Figure 4), correlations of H-1 (δ_H_ 2.99), H-5 (δ_H_ 2.32), H-10 (δ_H_ 2.43), and H-19 (δ_H_ 3.53) to C-17 (δ_C_ 166.4) suggested that C-17 was involved in the *N*=CH group, and correlations of H-5 (δ_H_ 2.32), H-6 (δ_H_ 4.45) to C-7 (δ_C_ 132.1), H-6 (δ_H_ 4.45), H-14 (δ_H_ 5.08), and H-15 (δ_H_ 4.80) to C-8 (δ_C_ 137.5) suggested the olefinic bond was located at C-7 and C-8, which supported the presence of skeleton of the 7,17-secoaconitine alkaloid. Moreover, HMBC correlation of H-14 (δ_H_ 5.08) to the carbonyl carbon signal of benzoyl group (δ_C_ 166.4) suggested that the benzoyl group was located at C-14; correlations of OCH_3_ (δ_H_ 3.20) to C-1 (δ_C_ 89.6), OCH_3_ (δ_H_ 3.19) to C-6 (δ_C_ 80.1), OCH_3_ (δ_H_ 3.75) to C-16 (δ_C_ 92.2), and OCH_3_ (δ_H_ 3.27) to C-18 (δ_C_ 80.6) suggested four methoxyl groups were linked at C-1, C-6, C-16 and C-18, respectively; correlations of H-10 (δ_H_ 2.43) and H-14 (δ_H_ 5.08) to C-13 (δ_C_ 75.6), H-7 (δ_H_ 5.62) and H-16 (δ_H_ 3.30) to C-15 (δ_C_ 74.1) suggested two hydroxyl group were linked at C-13 and C-15, respectively. Thus, the planar structure of **3** was deduced as 14-benzoyloxy-13,15-dihydroxy-1,6,16,18-tetramethoxy-7(8),17-dien-7,17-secoaconitane. Meanwhile, in the ROSEY spectrum (Figure 4) of **3**, the NOE correlations of H-1/H-10, H-10/H-14 and H-14/H-9 indicated *β*-orientation of H-1, H-9, H-10 and H-14, and α-axial configurations of 1-OCH_3_ and 14-benzoyloxy; the NOE correlations of H-6/H-5 and H-5/H-18 revealed β-orientation of H-18 and 18-OCH_3_, and α-axial of 6-OCH_3_; NOE correlations of H-17/H-16 and 15-OH, H-15/16-OCH_3_ revealed α-axial of H-16 and 15-OH, and β-orientation of 16-OCH_3_ and 13-OH. Moreover, the NOE correlations of H-1/H-3 and H-5 while no correlation between H-2 and H-5 indicated **3** had ring A (C-1, C-2, C-3, C-4, C-5, and C-11) in the chair conformation. Thus, according to the literature [18], compound **3** was assigned the name as (A-*c*)-14α-benzoyloxy-13β, 15α-dihydroxy-1α,6α,16β,18β-tetramethoxy-7(8),17-dien-7,17-secoaconitane.

**Figure 5:**
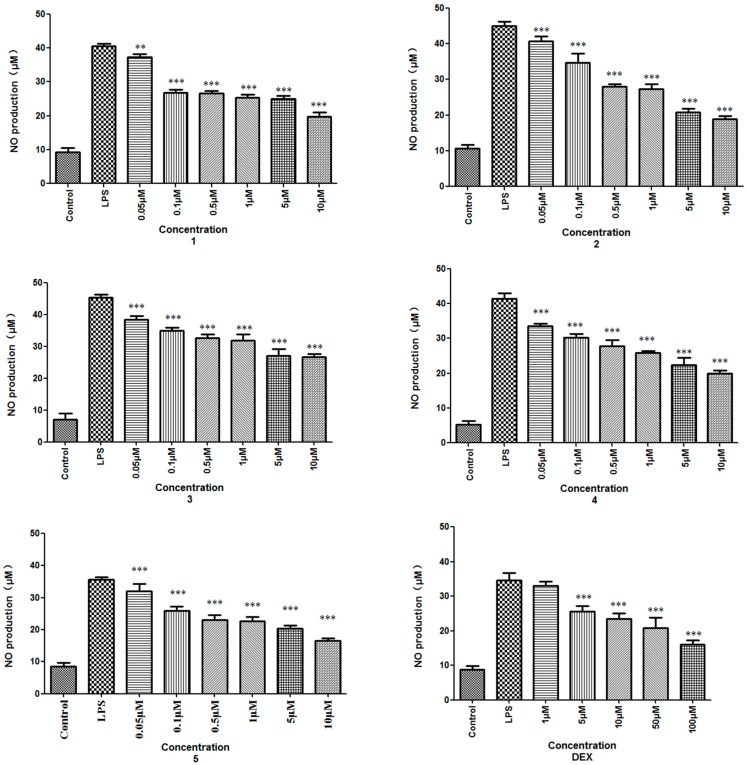
NO inhibitory effects of compounds from *A. szechenyianum* on LPS-activated RAW264.7 cells. Results represent the mean ± SD of three independent experiments; results differ significantly from the LPS-treated, ** *p* < 0.01, *** *p* < 0.001; dexamethasone (DEX) was used as a positive control.

Since the roots of *A. szechenyianum* are commonly used to treat rheumatism and fracture [17], in which inflammation is involved in the pathophysiological process and inhibitors of NO release are considered as potential anti-inflammatory agents for the treatment of these diseases [18,19,20], the isolated compounds from *A. szechenyianum* were evaluated using the Griess assay [21] for their effects on the inhibition of NO production in LPS-activated RAW264.7 cells. Dexamethasone (DEX) was selected as a positive control. As shown in Table 2 and Figure 5, all compounds with aconitine or 7,17-secoaconitine skeleton exhibited anti-inflammatory activities in a dose-dependent manner. Compared the activity with the substituent groups of **1**, **2**, **4**, and **5**, the structure-activity relationship may be due to the chemical environment of *N* atom. The compound **1** could hinder the inhibition of NO production with IC_50_ value of 36.62 ± 6.86 μM. The compound **2** exhibited excellent active performance with IC_50_ value of 3.30 ± 0.11 μM, indicated that the presence of *N*→O might increase anti-inflammatory activities. Moreover, compound **4** exhibited effective inhibitory activity with IC_50_ value of 8.09 ± 1.31 μM and compound **5** showed inhibitory activity with IC_50_ value of 11.73 ± 1.94 μM. In addition, compound **3** as a 7,17-secoaconitine type alkaloid also exhibited potent inhibitory activity on NO production with IC_50_ value of 7.46 ± 0.89 μM.

## 3. Experimental Section

### 3.1. General Information

ESI-MS was performed on a Quattoro Premier instrument (Waters, Milford, MA, USA). The HR-ESI-MS spectra were recorded on an Agilent Technologies 6550 Q-TOF (Santa Clara, CA, USA). 1D and 2D-NMR spectra were recorded on Bruker-AVANCE 400 instrument (Bruker, Rheinstetten, Germany) with TMS as an internal standard. The analytical HPLC was performed on a Waters e2695 Separations Module coupled with a 2998 Photodiode Array Detector and a Accurasil C-18 column (4.6 mm × 250 mm, 5 μm particles, Ameritech, Chicago, IL, USA). Semipreparative HPLC was performed on a system comprising an LC-6AD pump equipped with an SPD-20A UV detector (Shimadzu, Kyoto, Japan) and an Ultimate XB-C18 (10 mm × 250 mm, 5 μm particles) or YMS-Pack-ODS-A (10 mm × 250 mm, 5 μm particles). Silica gel was purchased Qingdao Haiyang Chemical Group Corporation (Qingdao, China).

### 3.2. Plant Material

The roots of *Aconitum szechenyianum* Gay. were collected from the Xi Mountains of Gansu Province of China in July 2014, and identified by senior experimentalist Jitao Wang. A voucher specimen (herbarium No. 20140728) has been deposited in the Medicinal Plants Herbarium (MPH), Shaanxi University of Chinese Medicine, Xianyang, China.

### 3.3. Extraction and Isolation

The air-dried and powdered underground parts of *A. szechenyianum* (5.0 kg) were extracted with 80% EtOH at 80 °C for three times (each time 40 L for 1.5 h). After removal of EtOH solvent under reduced pressure, the extract (2 L) was dispersed in water (1.5 L), adjusted with 9% HCl solution to pH 0.8, and extracted with petroleum ether (PE). The acidic water solution was alkalized to pH 10.26 with 25% ammonia solution, extracted with CHCl_3_ three times, and evaporated under pressure to give crude alkaloids (50 g). The crude alkaloids (47 g) were chromatographed on silica gel column, eluting with gradient solvent system (PE/acetone/diethylamine, 50:1:0.1–1:1:0.1) to give 12 fractions (Fr.1–Fr.12). Fr.6 (3.2 g) was purified by HPLC (YMC-Pack-ODS-A, 10 mm × 250 mm, 5 μm particles, flow rate: 1.0 mL·min^−1^) with CH_3_OH/H_2_O (70:30) as mobile phase to obtained Fr.6-1 (30 mg; *t_R_* = 5 min), Fr.6-2 (120 mg; *t_R_* = 30 min), Fr.6-3 (130 mg; *t_R_* = 42 min), Fr.6-4 (120 mg; *t_R_* = 63 min), Fr.6-5 (140 mg; *t_R_* = 77 min), and Fr.6-6 (200 mg; *t_R_* = 63 min). Fr.6-3 (130mg) was purified by HPLC with CH_3_OH/H_2_O (60:40) as mobile phase to afford **1** (13 mg; *t_R_* = 50 min) and **2** (12 mg; *t_R_* = 65 min). Fr.6-4 (120 mg) was purified by HPLC with CH_3_OH/H_2_O (65:35) as mobile phase to afford **3** (10 mg; *t_R_* = 45 min), **4** (20 mg; *t_R_* = 65 min), and **5** (25 mg; *t_R_* = 75 min). See more detailed spectrums in the supplementary materials.

*(A-c)-14α-Benzoyloxy-8β-acetoxyl-13β,15α-dihydroxy-1α,6α,16β,18β-tetramethoxy-19-en-aconitane* (*szechenyianine* A): A white amorphous powder, ${\left[\alpha \right]}_{D}^{20}$ +60.2 (*c* 0.38, MeOH), IR (KBr) ν_max_: 3508, 2936, 1718, 1637 and 714 cm^−1^; ^1^H-NMR (400 MHz, CDCl_3_) and ^13^C-NMR (100 MHz, CDCl_3_) spectral data, see Table 1; *m/z* 600.2842 [M + H]^+^ (calcd. for C_32_H_41_NO_10_, 600.2809).

*(A-c)-14α-Benzoyloxy-8β-acetoxyl-13β,15α-dihydroxy-1α,6α,16β,18β-tetramethoxy-19-en-aconitane-N-oxide (szechenyianine B)*: A white amorphous powder, [α]${}_{D}^{20}$ +10.5 (*c* 0.44, MeOH), IR (KBr) ν_max_: 3510, 2938, 1719, 1603 and 716 cm^−1^; ^1^H-NMR (400 MHz, CDCl_3_) and ^13^C-NMR (100 MHz, CDCl_3_) spectral data, see Table 1; *m/z* 616.2783 [M + H]^+^ (calcd. for C_32_ H_41_NO_11_, 616.2758).

*(A-c)-14α-Benzoyloxy-13β,15α-dihydroxy-1α,6α,16β,18β-tetramethoxy-7(8),17-dien-7,17-secoaconitane (szechenyianine C)*: A white amorphous powder, [α]${}_{D}^{20}$ +21.6 (*c* 0.64, MeOH), IR (KBr) ν_max_: 3513, 2930, 2824, 1716, 1645, 1453, 1367, 1275, 1102 and 715 cm^−1^; ^1^H-NMR (400 MHz, CDCl_3_) and ^13^C-NMR (100 MHz, CDCl_3_) spectral data, see Table 1; *m/z* 542.2783 [M + H]^+^ (calcd. for C_30_ H_39_NO_8_, 542.2754).

### 3.4. Inhibitory Assay of NO Production

Assays for NO production were carried out according to the Griess reaction, using dexamethasone as positive control. Briefly, RAW264.7 cells were seeded into 96-well microplates at a density of 2 × 10^5^ mL^−1^ and allowed to adhere for 4 h. RPMI1640 (100 μL) containing test samples (final concentration of 10, 5, 1, 0.5, 0.1, and 0.05 μM) dissolved in DMSO (final concentration less than 0.2%) and LPS (final concentration of 1 μg·mL^−1^) were added. After incubation at 37 °C for 18 h, 50 μL of cell-free supernatant was mixed with 50 μL of Griess Reagent I and 50 μL of Griess Reagent II to determine NO production. Absorbance was measured at 550 nm against a calibration curve with NaNO_2_ standard. The NO productions of the isolated compounds were tested (Figure 5), the inhibitory rate on NO production induced by LPS was calculated by the NO_2_^−^ levels as follows: Inhibitory rate (%) = 100 × ([NO_2_^−^]_LPS_ − [NO_2_^−^]_LPS+sample_)/([NO_2_^−^]_LPS_ − [NO_2_^−^]_untreated_), the IC_50_ values were calculated (Table 2). Values are mean ± SD, *n* = 3, ** *p* < 0.01, *** *p* < 0.001 vs. LPS treated.

## Figures and Tables

**Table 1:** ^1^H-NMR and ^13^C-NMR spectral data of compounds **1**–**5**.

NO.	1		2		3		4	5
	δ_C_	δ_H_ (*J* in Hz)	δ_C_	δ_H_ (*J* in Hz)	δ_C_	δ_H_ (*J* in Hz)	δ_C_	δ_C_
1	82.3	3.20 (d, 4.1)	80.5	3.32 (m)	89.6	2.99 (dd, 4.4, 11.3)	81.0	82.3
2	22.9	1.66 (m, H-2a)	22.0	1.45 (m, H-2a)	24.7	1.10 (m)	31.8	23.5
		1.57 (m, H-2b)		1.81 (m, H-2b)		1.86 (m)		
3	28.2	1.63 (m, H-3a)	29.5	1.70 (m, H-3a)	37.4	1.55 (m)	72.3	29.0
		1.64 (m, H-3b)		1.79 (m, H-3b)		1.69 (m)		
4	46.8		42.1		39.7		43.2	39.0
5	45.8	2.23 (d, 7.1)	44.7	2.35 (d, 7.0)	46.2	2.32 (d, 8.9)	51.2	48.7
6	84.1	3.92 (d, 7.1)	82.7	4.00 (d, 7.0)	80.1	4.45 (m)	83.8	83.2
7	49.6	2.87 (s)	49.7	3.30 (s)	132.1	5.62 (d, 5.5)	45.2	43.6
8	90.6		89.3		137.5		91.7	91.5
9	42.6	2.70 (t, 6.1)	41.9	2.74 (m)	43.0	3.18 (s)	43.9	43.2
10	40.6	2.17 (m)	39.1	2.26 (m)	41.7	2.43 (s)	40.9	40.3
11	51.4		51.9		48.5		49.4	49.7
12	36.4	2.20 (m, H-12a)	36.5	2.27 (m, H-12a)	38.9	2.45 (m)	35.2	35.4
		2.21 (m, H-12b)		1.98 (m, H-12b)				
13	74.3		74.1		75.6		74.4	74.0
14	79.3	4.90 (d, 4.9)	78.8	4.89 (d, 4.8)	79.4	5.08 (d, 4.2)	79.1	78.9
15	78.9	4.48 (dd, 2.9, 5.3)	78.7	4.48 (dd, 3.0, 4.9)	74.1	4.80 (dd, 3.0, 5.8)	79.2	79.0
16	89.9	3.42 (d, 5.3)	89.6	3.45 (d, 5.0)	92.2	3.30 (d, 6.0)	90.0	89.5
17	60.6	3.97 (s)	72.9	4.02 (s)	166.4	7.86 (br s)	55.8	56.7
18	78.2	3.78 (d, 8.5, H-18a)	77.9	3.79 (d, 8.5, H-18a)	80.6	3.16 (d, 8.4)	73.8	79.8
		3.42 (d, 8.5, H-18b)		3.33 (d, 8.5, H-18b)		3.86 (d, 8.4)		
19	165.9	7.31 (s)	138.9	6.70 (d, 1.2)	58.3	3.53 (m)	41.6	49.0
						3.45 (m)		
8-OAc	172.6		172.1				172.3	172.0
	21.5	1.32 (s)	21.4	1.32 (s)			21.5	21.3
1-OCH_3_	56.3	3.18 (s)	56.6	3.21 (s)	58.2	3.20 (s)	56.0	55.4
6-OCH_3_	57.4	3.03 (s)	57.3	3.05 (s)	56.9	3.19 (s)	58.3	57.9
16-OCH_3_	61.3	3.75 (s)	61.4	3.77 (s)	61.8	3.75 (s)	61.4	61.1
18-OCH_3_	59.3	3.29 (s)	59.3	3.27 (s)	59.1	3.27 (s)	59.1	59.1
ArC=O	166.2		166.2		166.4		166.2	165.9
ArC-1′	130.0		129.9		130.0		130.0	130.7
3′, 5′	128.9	7.43 (t, 7.6)	129.0	7.44 (t, 7.3)	128.7	7.42 (t, 7.5)	128.9	128.6
2′, 6′	129.8	8.02 (d, 7.6)	129.9	8.01 (d, 7.3)	130.1	8.03 (d, 7.5)	129.8	129.6
4′	133.6	7.55 (t, 7.6)	133.8	7.57 (t, 7.3)	133.5	7.53 (t, 7.5)	133.5	133.3

δ in CDCl_3,_ in ppm from TMS; coupling constants (*J*) in Hz; ^1^H-NMR at 400 MHz and ^13^C-NMR at 100 MHz.

**Table 2:** IC_50_ values of the compounds from *A. szechenyianum* on NO production in LPS-activated RAW264.7 cells.

Compound	1	2	3	4	5	Dexamethasone
IC_50_ (μM)	36.62 ± 6.86	3.30 ± 0.11	7.46 ± 0.89	8.09 ± 1.31	11.73 ± 1.94	8.32 ± 1.45

Results are expressed as IC_50_ values in μM and the values are means ± SD; *n* = 3; dexamethasone was used as a positive control.

## Supplementary Materials

IR, HR-ESI-MS, ^1^H-NMR, ^13^C-NMR and 2D NMR spectra for compounds **1**–**3** can be found, in the online version, at http://www.mdpi.com/1420-3049/21/9/1175/s1.
