# Supplementary material for: C19-Norditerpenoid Alkaloids from Aconitum szechenyianum and Their Effects on LPS-Activated NO Production
Source: Molecules. 2016 Sep 3;21(9):1175. doi: 10.3390/molecules21091175 (PMC6273406; doi:10.3390/molecules21091175)
Supplement: Supplementary file 1 [file molecules-21-01175-s001.pdf]

Supplementary Materials: C19-Norditerpenoid Alkaloids from *Aconitum szechenyianum* and Their Effects on LPS-Activated NO Production

Fei Wang, Zhenggang Yue, Pei Xie, Li Zhang, Zhen Li, Bei Song, Zhishu Tang and Xiaomei Song

List of Content

No.	Content	Page
1	**Figure S1.** The HR-ESI-MS spectrum of **1** (in MeOH).	S2
2	**Figure S2.** The IR spectrum of **1** (in KBr).	S3
3	**Figure S3.** The ^{1}H NMR spectrum of **1** (400MHz in $CDCl_3$).	S4
4	**Figure S4.** The ^{13}C NMR spectrum of **1** (100MHz in $CDCl_3$).	S5
5	**Figure S5.** The HSQC spectrum of **1** (400MHz in $CDCl_3$).	S6
6	**Figure S6.** The ^{1}H-^{1}H COSY spectrum of **1** (400MHz in $CDCl_3$).	S7
7	**Figure S7.** The HMBC spectrum of **1** (400MHz in $CDCl_3$).	S8
8	**Figure S8.** The ROESY spectrum of **1** (400MHz in $CDCl_3$).	S9
9	**Figure S9.** The HR-ESI-MS spectrum of **2** (in MeOH).	S10
10	**Figure S10.** The IR spectrum of **2** (in KBr).	S11
11	**Figure S11.** The ^{1}H NMR spectrum of **2** (400MHz in $CDCl_3$).	S12
12	**Figure S12.** The ^{13}C NMR spectrum of **2** (100MHz in $CDCl_3$).	S13
13	**Figure S13.** The HSQC spectrum of **2** (400MHz in $CDCl_3$).	S14
14	**Figure S14.** The ^{1}H-^{1}H COSY spectrum of **2** (400MHz in $CDCl_3$).	S15
15	**Figure S15.** The HMBC spectrum of **2** (400MHz in $CDCl_3$).	S16
16	**Figure S16.** The ROESY spectrum of **2** (400MHz in $CDCl_3$).	S17
17	**Figure S17.** The HR-ESI-MS spectrum of **3** (in MeOH).	S18
18	**Figure S18.** The IR spectrum of **3** (in KBr).	S19
19	**Figure S19.** The ^{1}H NMR spectrum of **3** (400MHz in $CDCl_3$).	S20
20	**Figure S20.** The ^{13}C NMR spectrum of **3** (100MHz in $CDCl_3$).	S21
21	**Figure S21.** The HSQC spectrum of **3** (400MHz in $CDCl_3$).	S22
22	**Figure S22.** The ^{1}H-^{1}H COSY spectrum of **3** (400MHz in $CDCl_3$).	S23
23	**Figure S23.** The HMBC spectrum of **3** (400MHz in $CDCl_3$).	S24
24	**Figure S24.** The ROESY spectrum of **3** (400MHz in $CDCl_3$).	S25

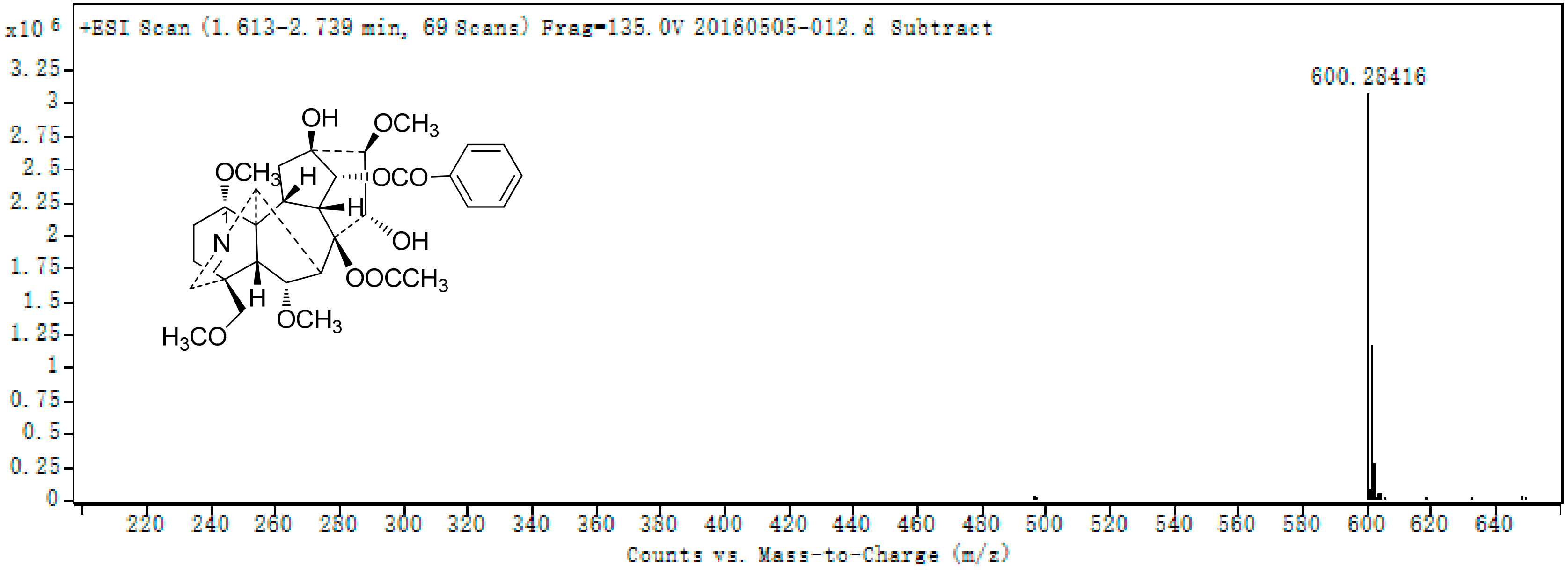

Figure S1. The HR-ESI-MS spectrum of **1** (in MeOH).

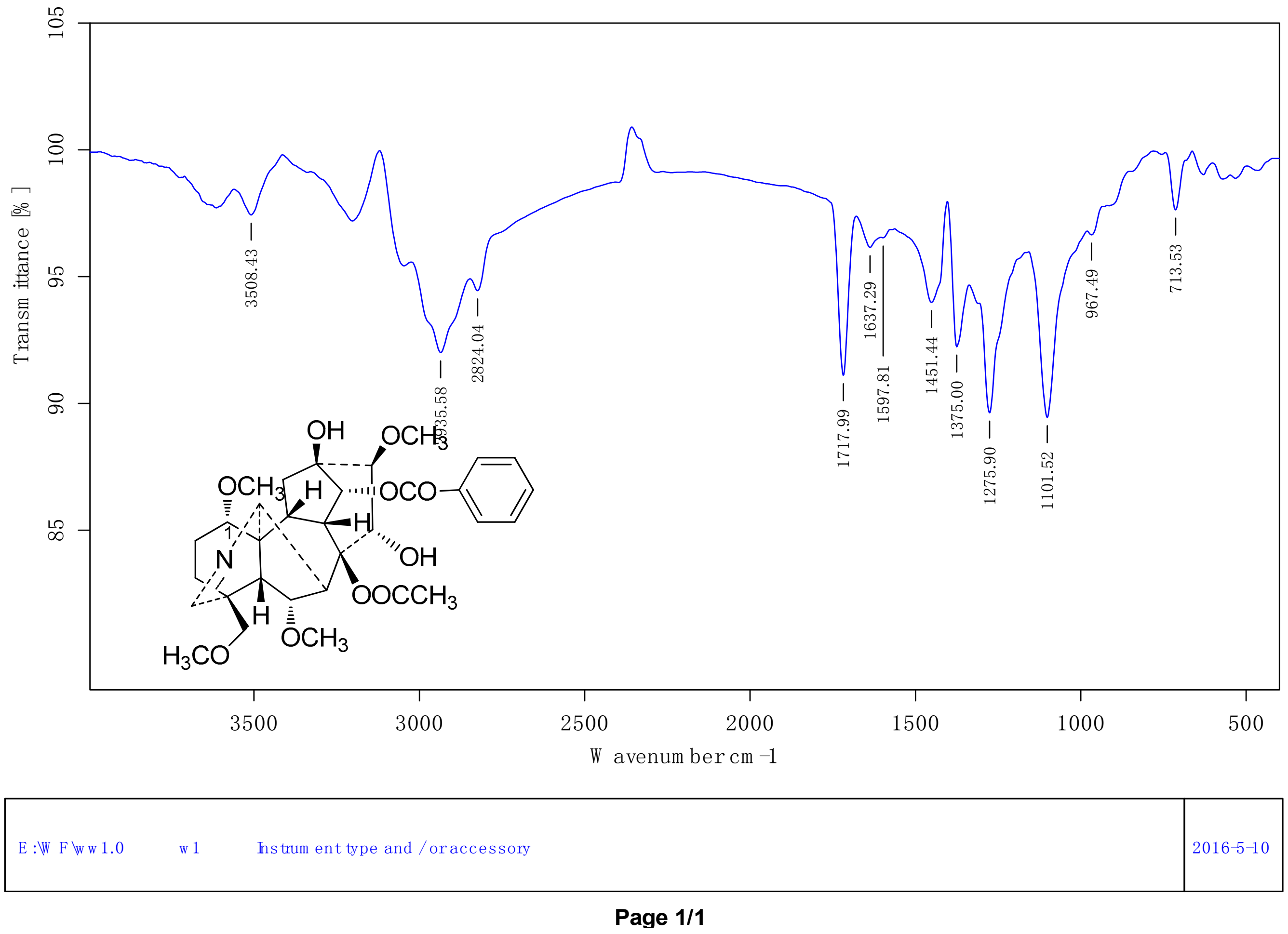

Figure S2. The IR spectrum of **1** (in KBr).

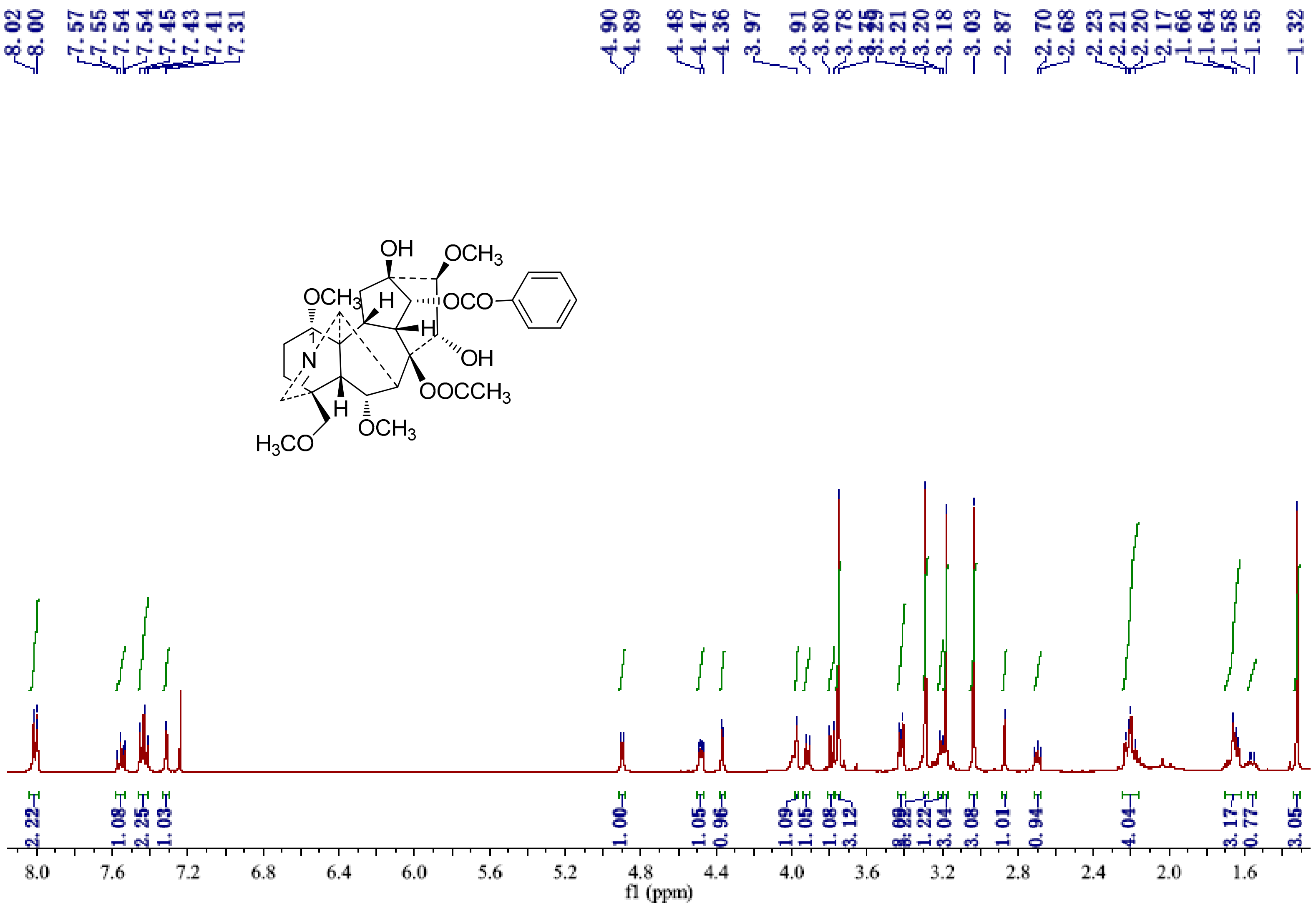

Figure S3. The ^{1}H-NMR spectrum of **1** (400MHz in $CDCl_3$).

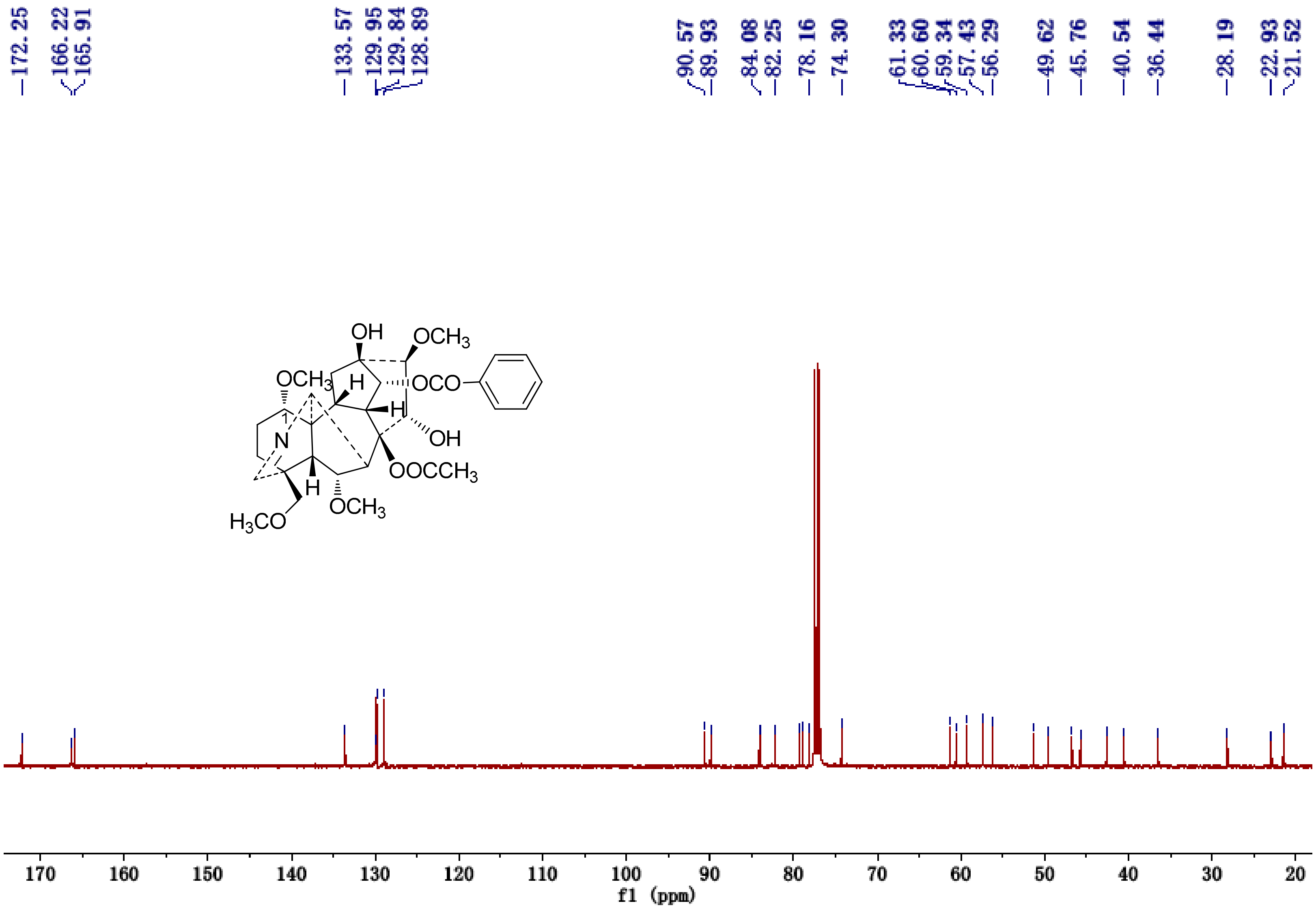

Figure S4. The ^{13}C-NMR spectrum of **1** (100MHz in $CDCl_3$).

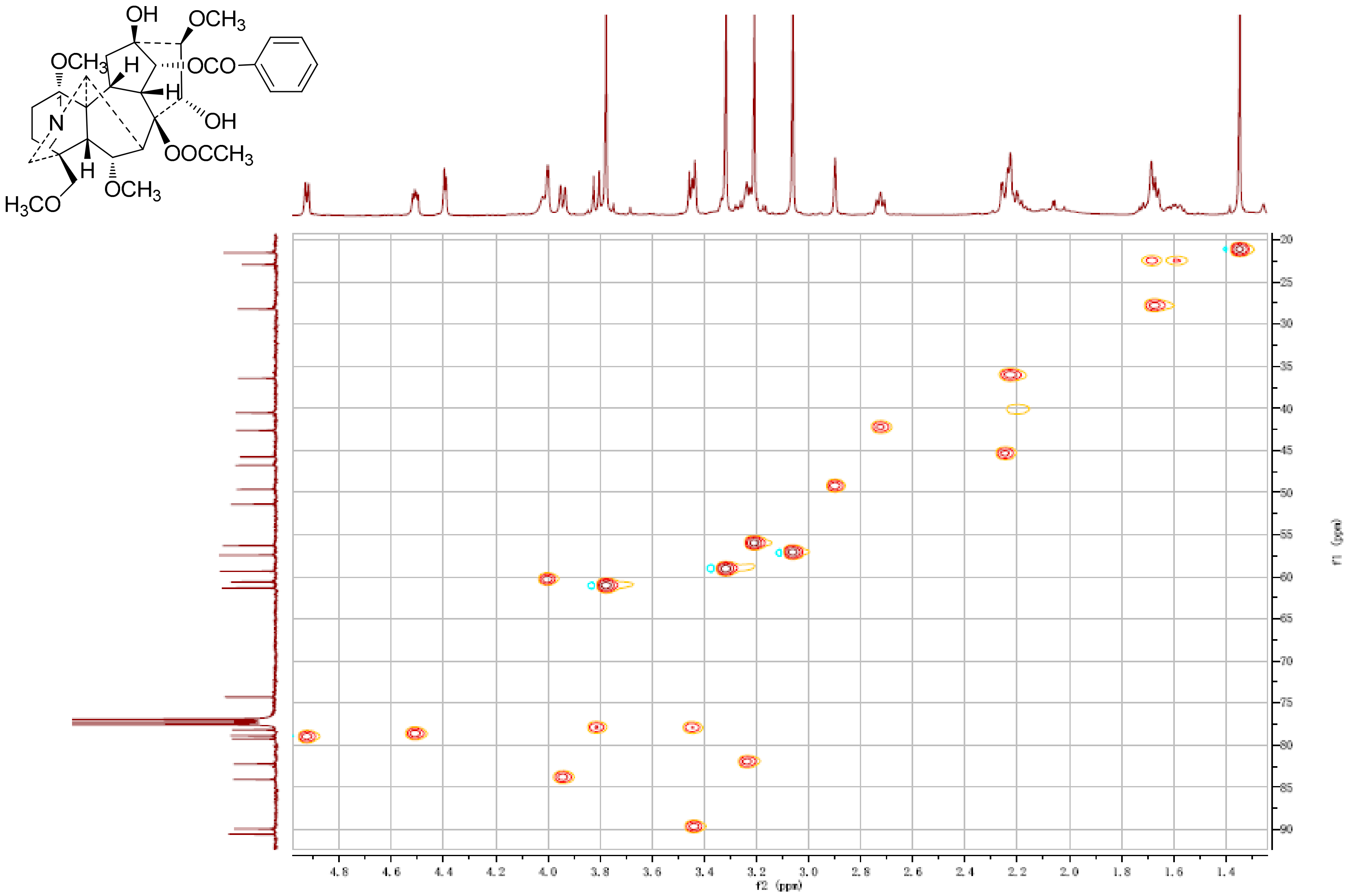

Figure S5. The HSQC spectrum of **1** (400MHz in CDCl3).

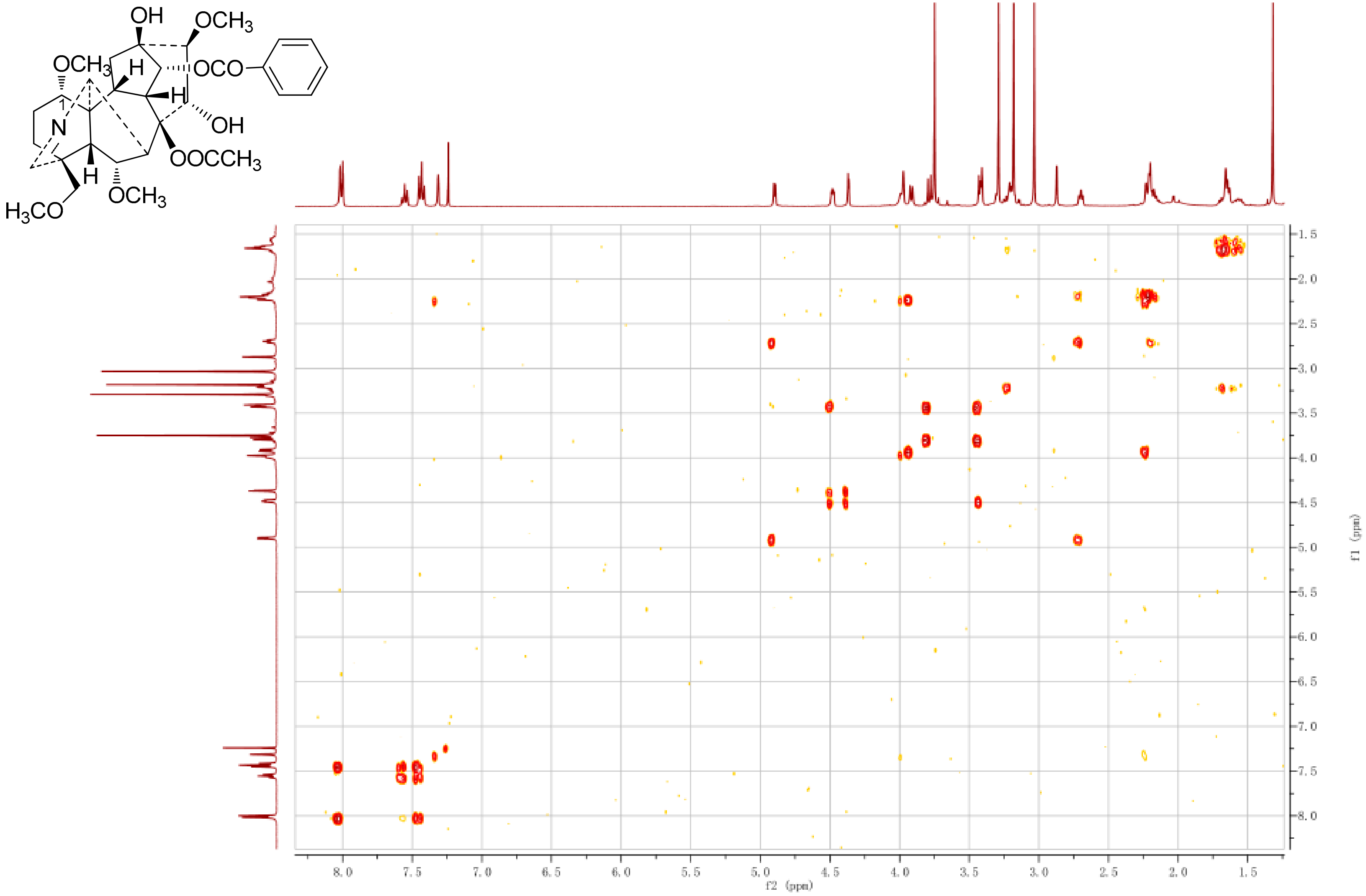

Figure S6. The ^1H-^1H COSY spectrum of **1** (400MHz in $CDCl_3$).

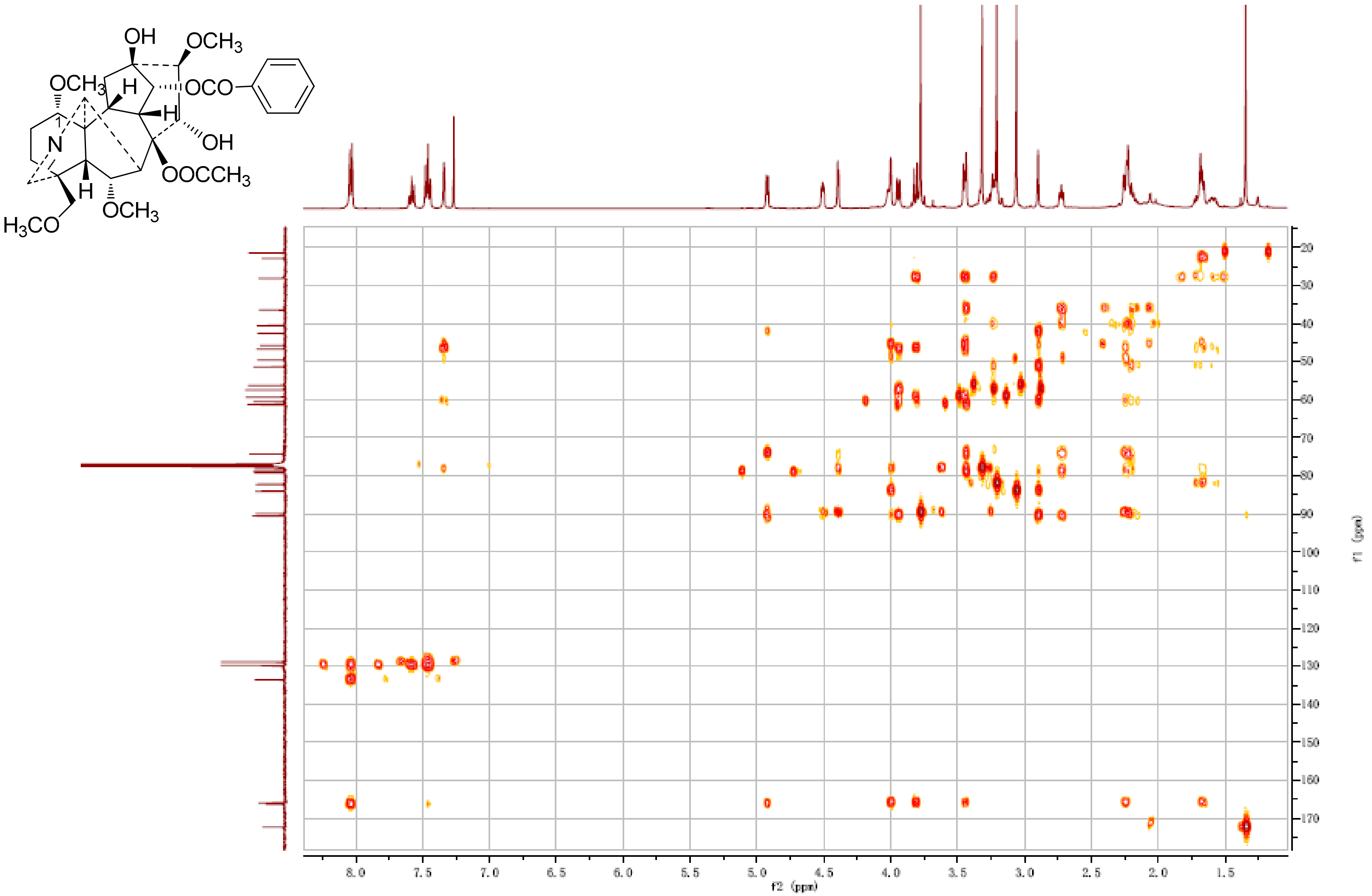

Figure S7. The HMBC spectrum of **1** (400MHz in $CDCl_3$).

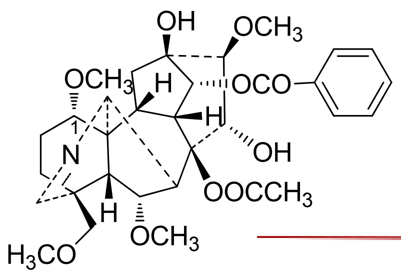

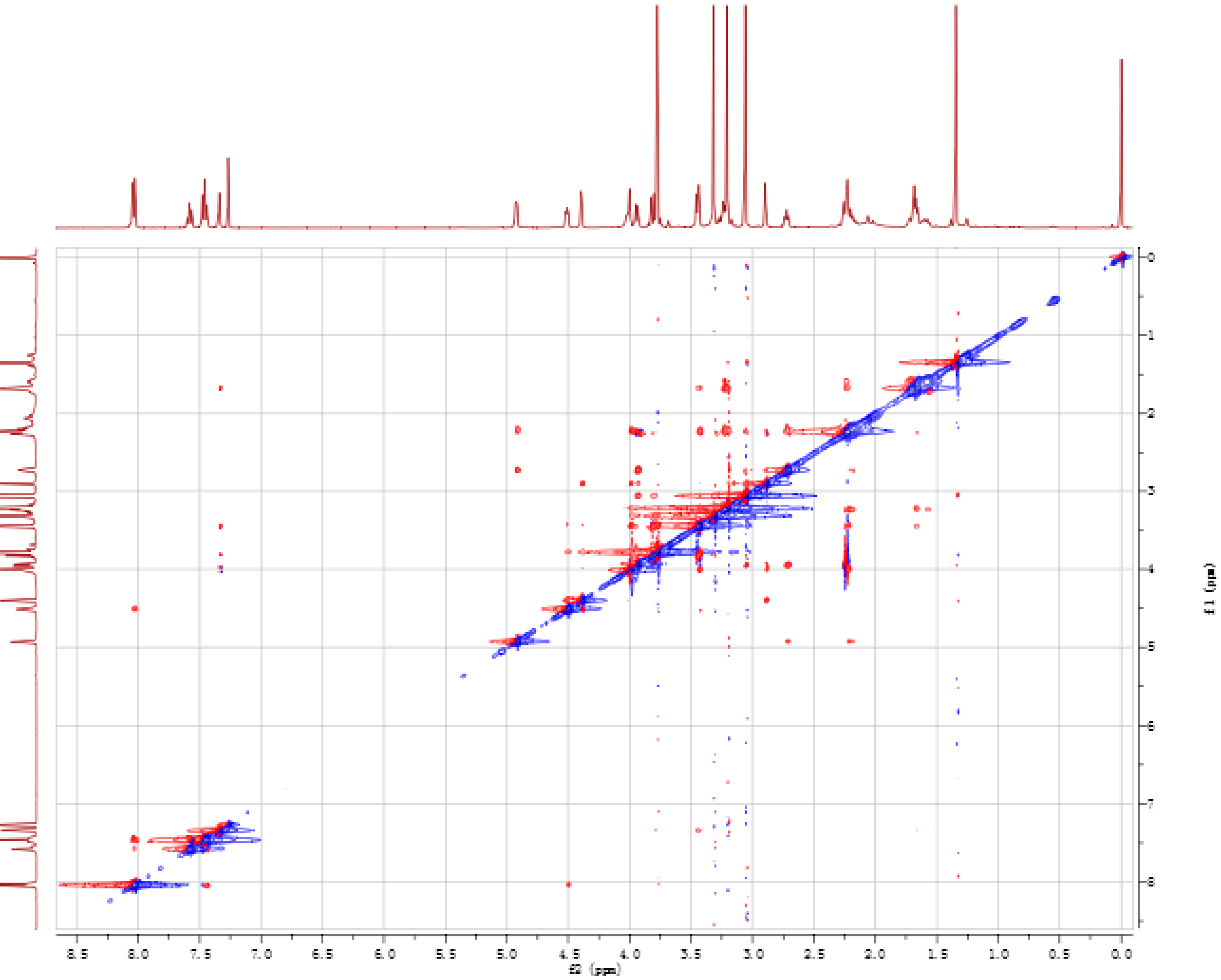

Figure S8. The ROESY spectrum of **1** (400MHz in $CDCl_3$).

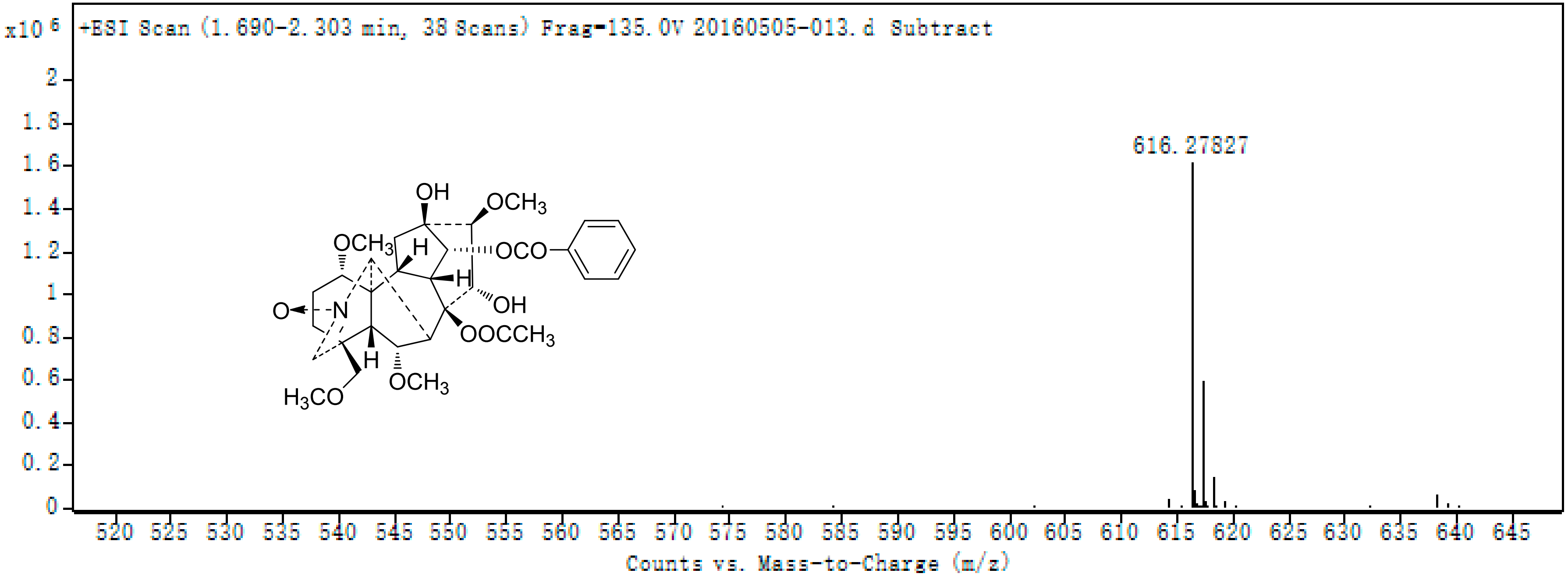

Figure S9. The HR-ESI-MS spectrum of 2 (in MeOH).

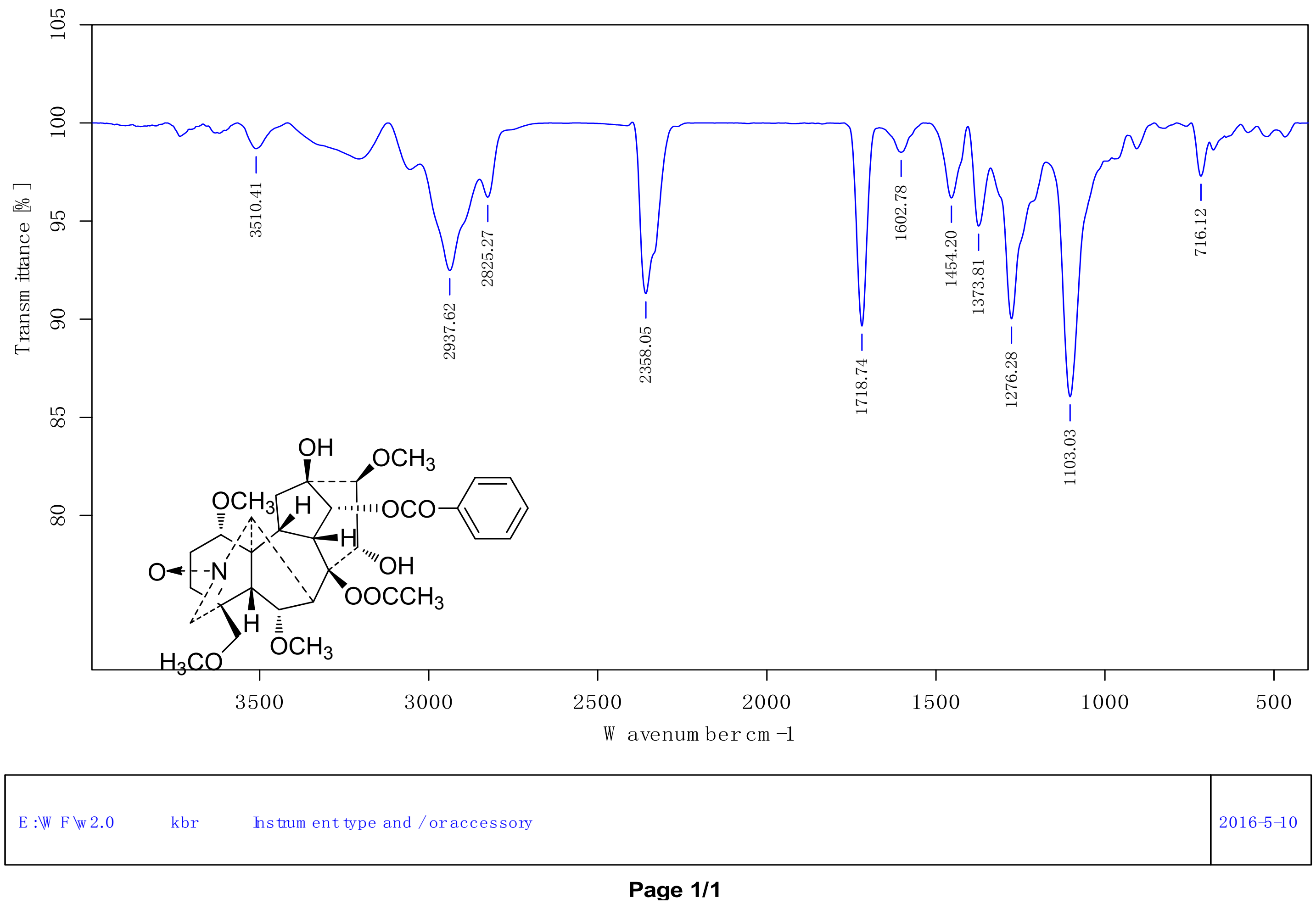

Figure S10. The IR spectrum of **2** (in KBr).

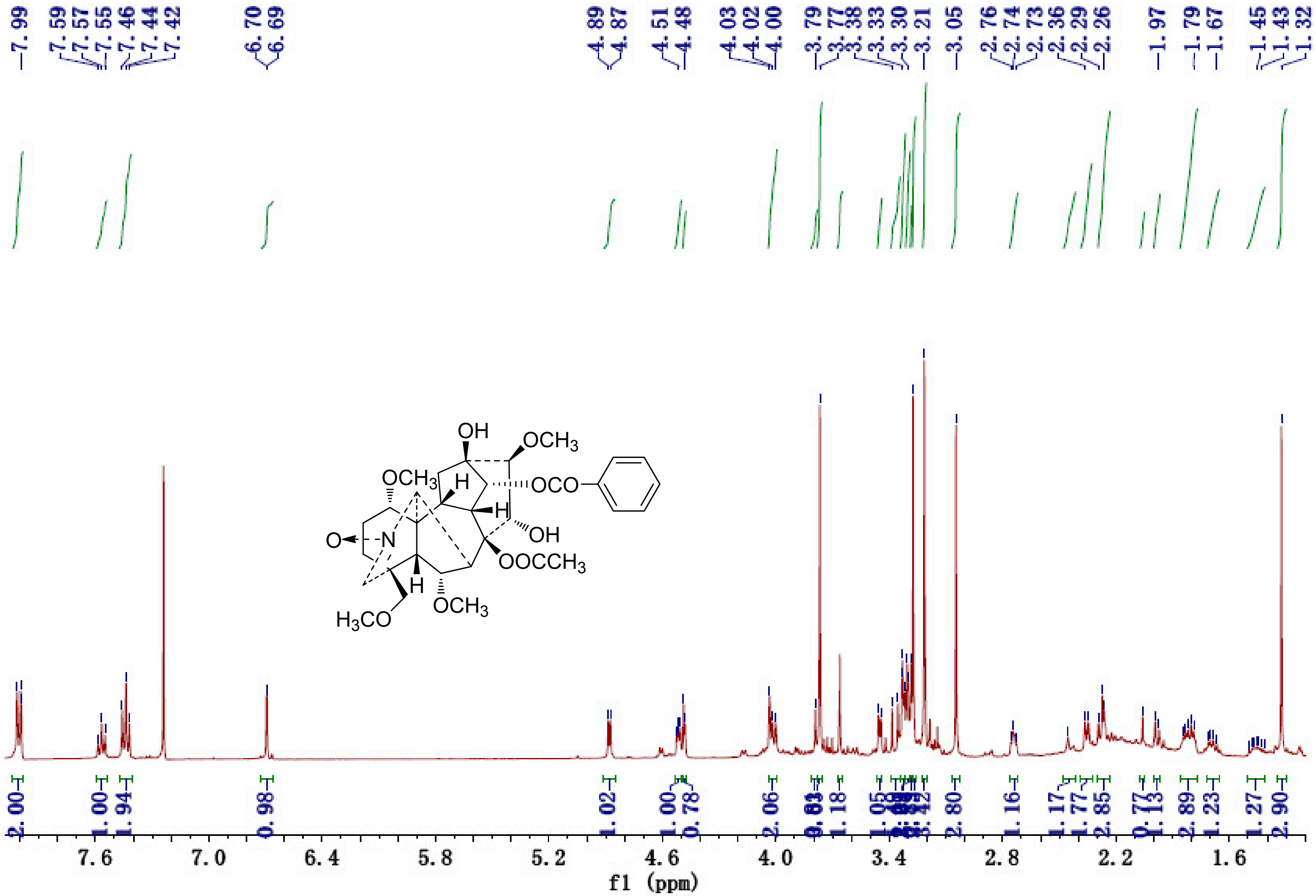

Figure S11. The ^{1}H-NMR spectrum of **2** (400 MHz in $CDCl_3$).

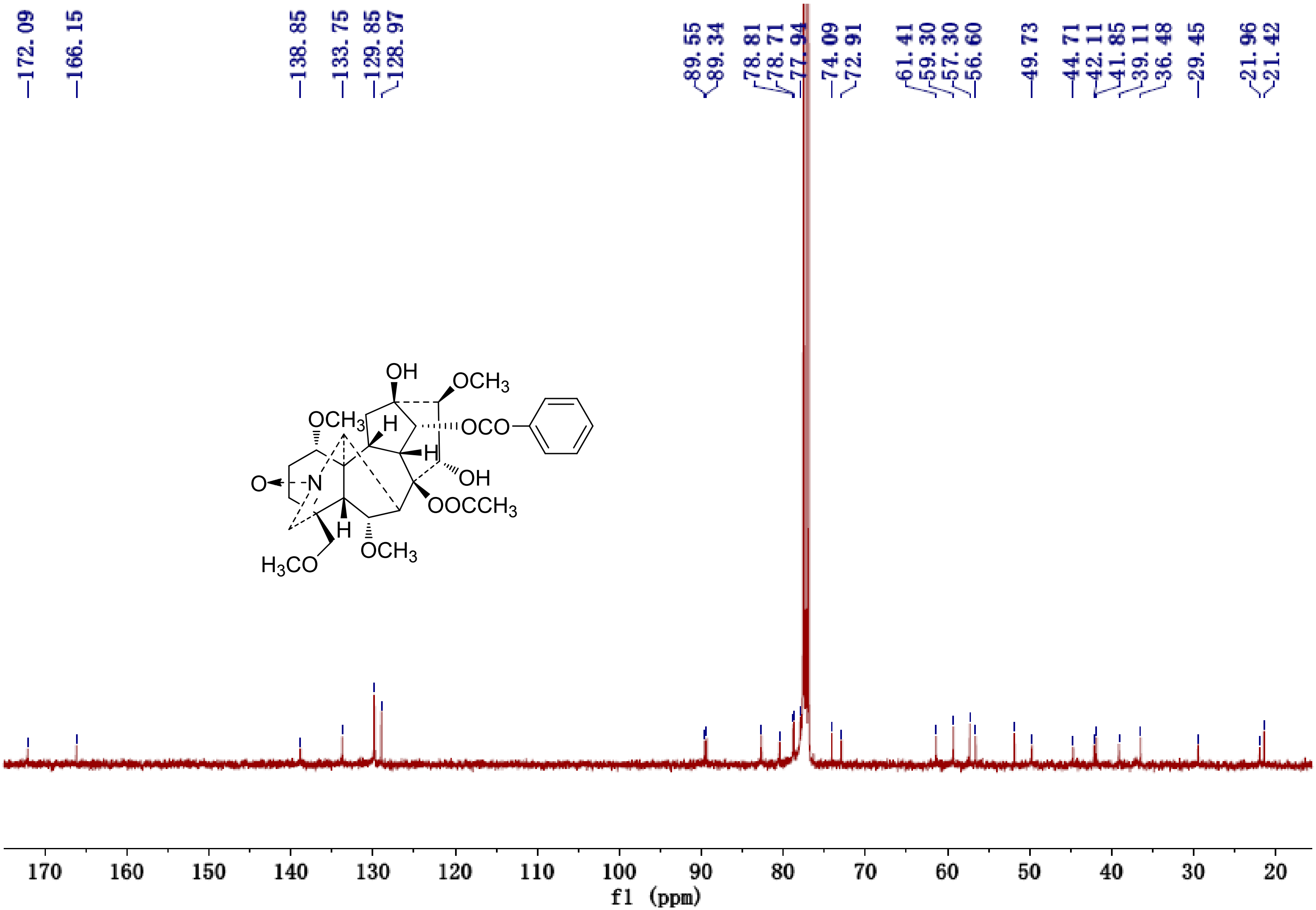

Figure S12. The ^{13}C-NMR spectrum of **2** (100MHz in $CDCl_3$).

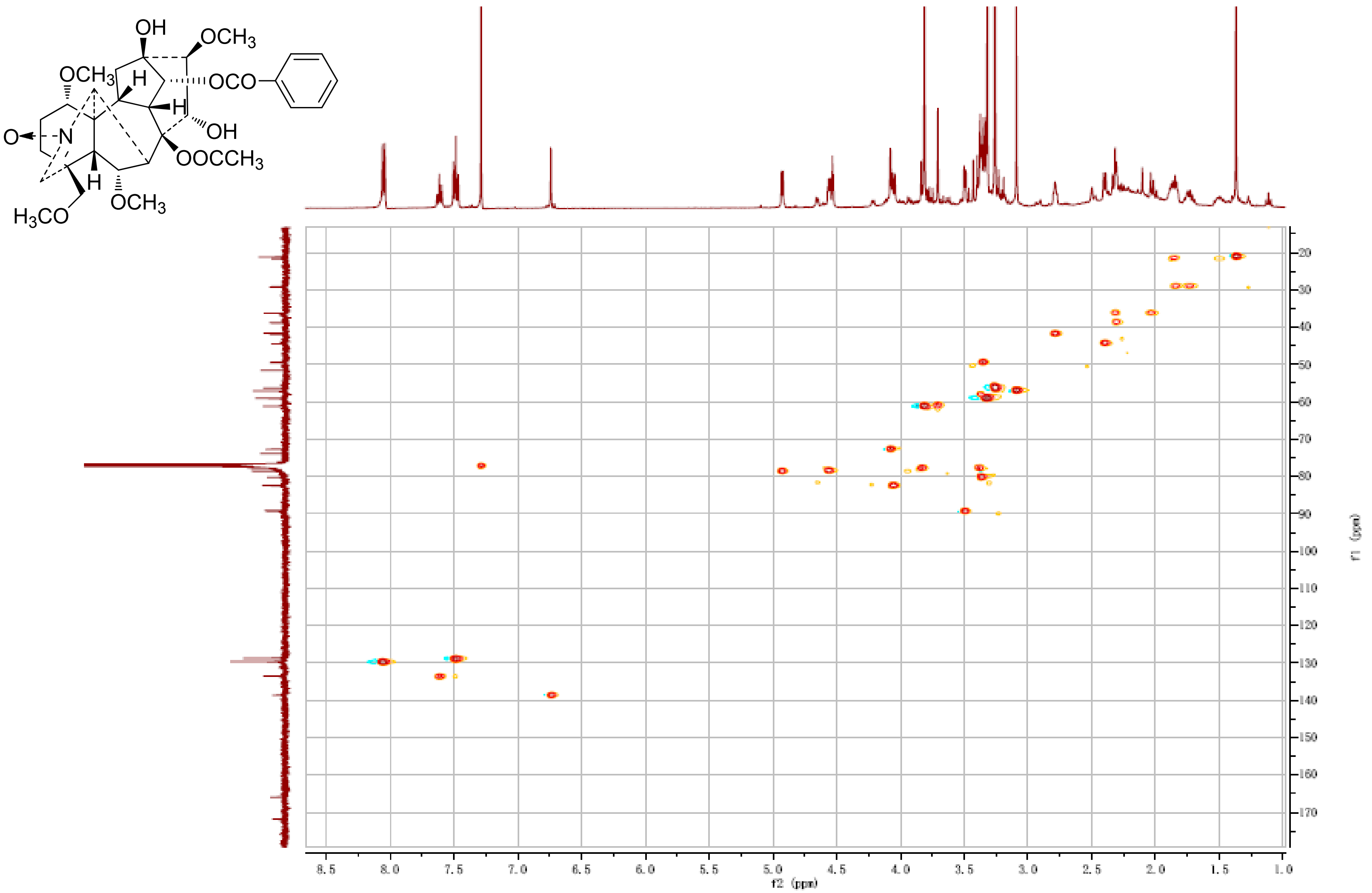

Figure S13. The HSQC spectrum of **2** (400MHz in $CDCl_3$).

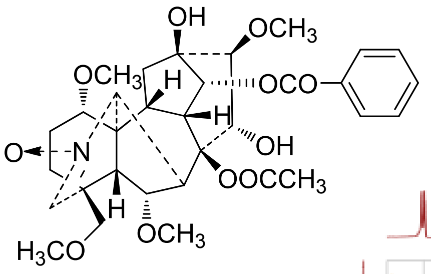

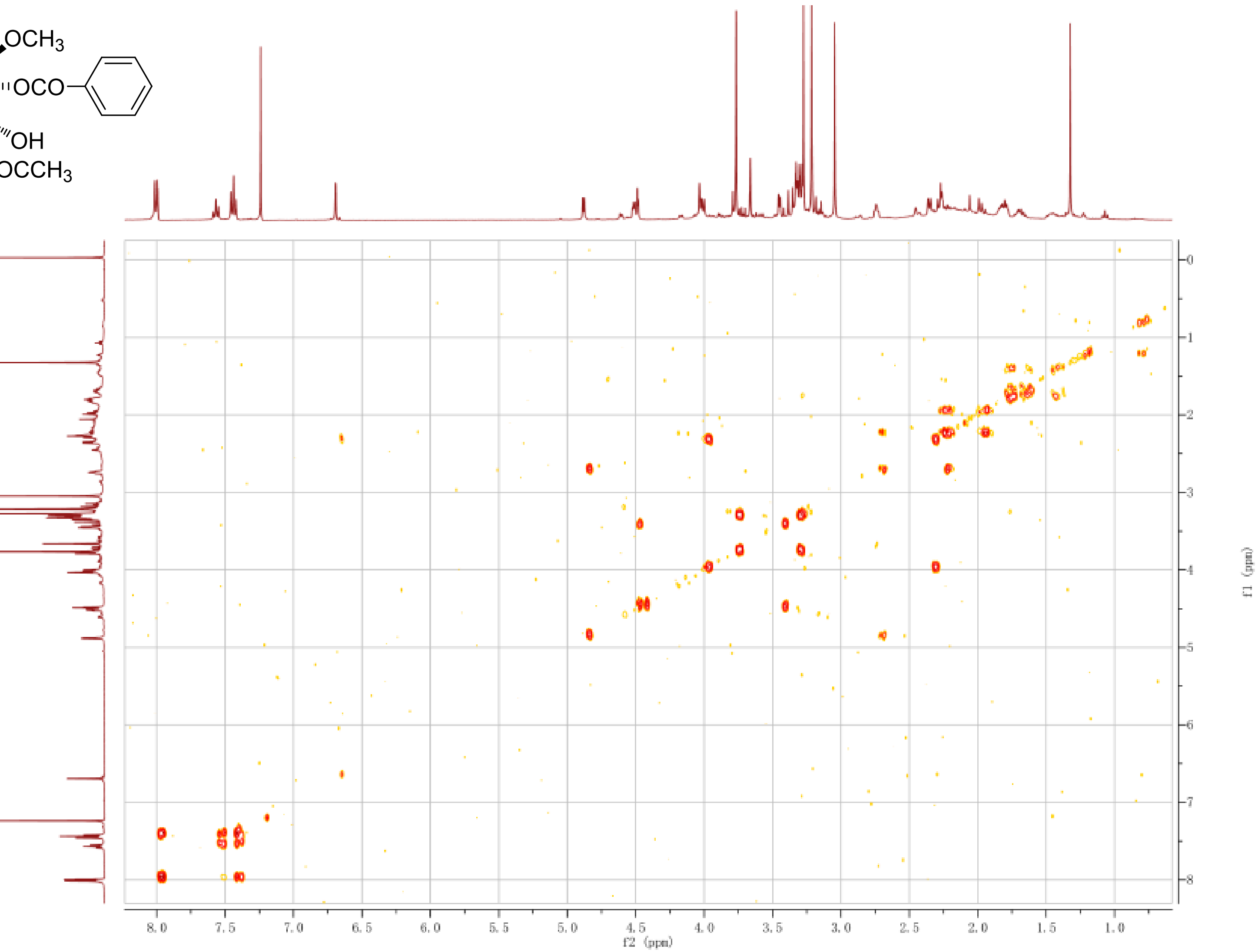

Figure S14. The ^{1}H-^{1}H COSY spectrum of **2** (400MHz in $CDCl_3$).

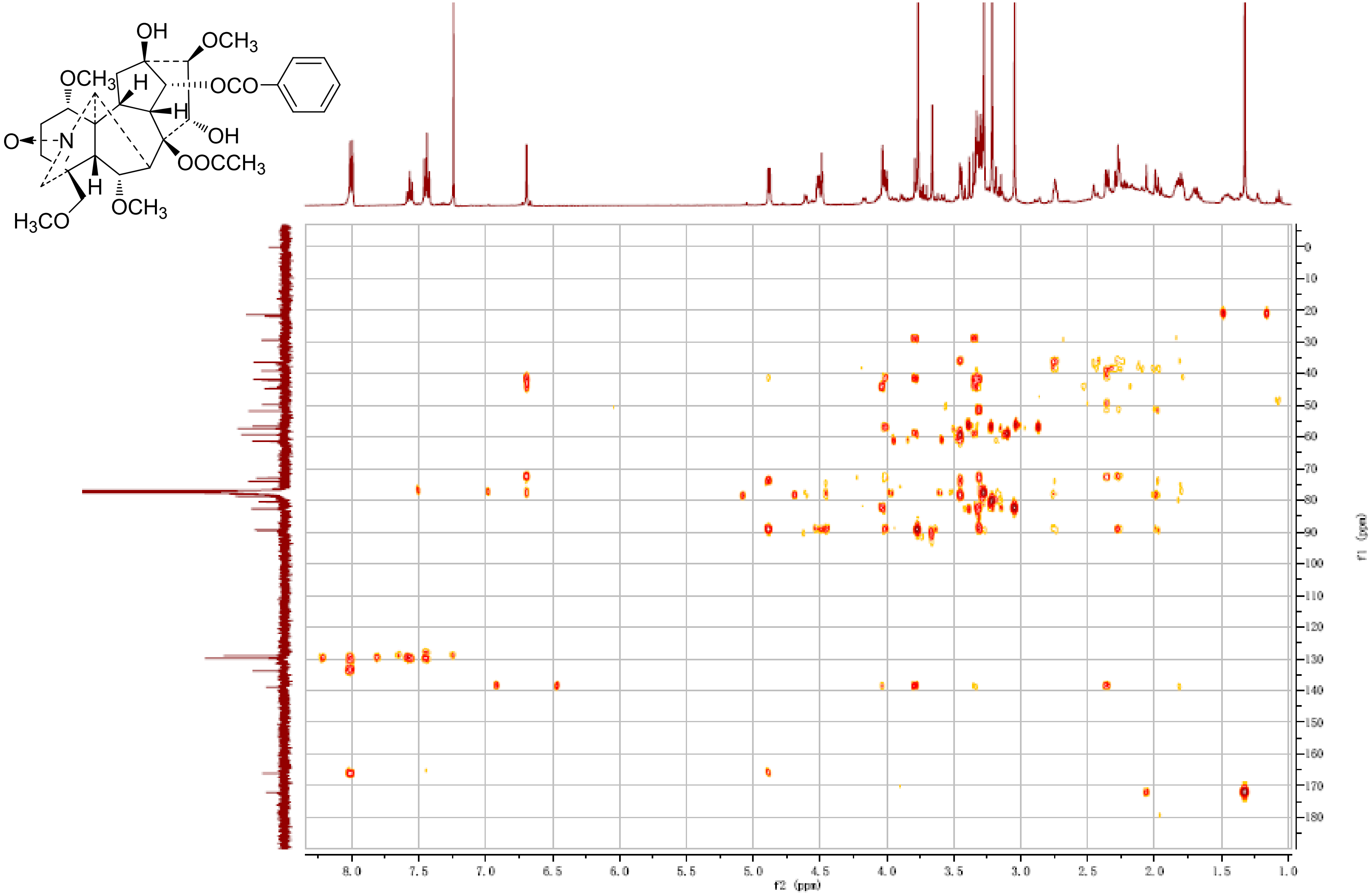

Figure S15. The HMBC spectrum of **2** (400MHz in CDCl3).

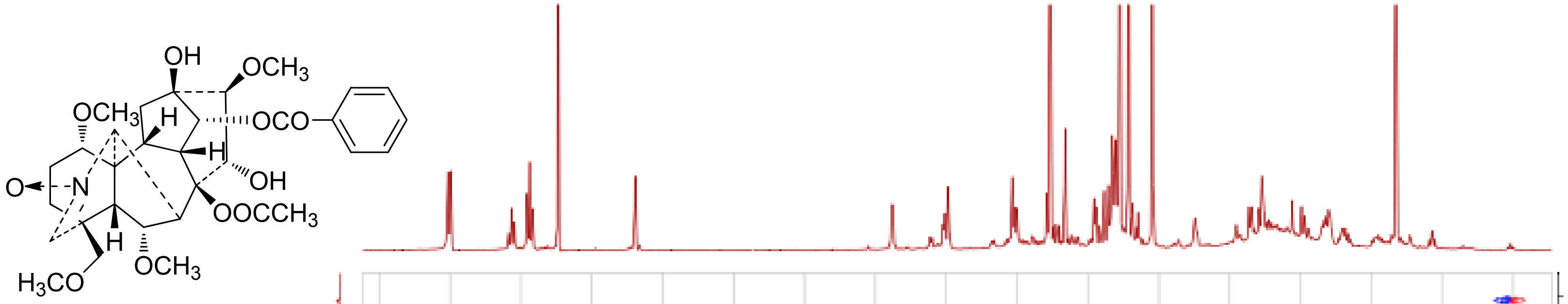

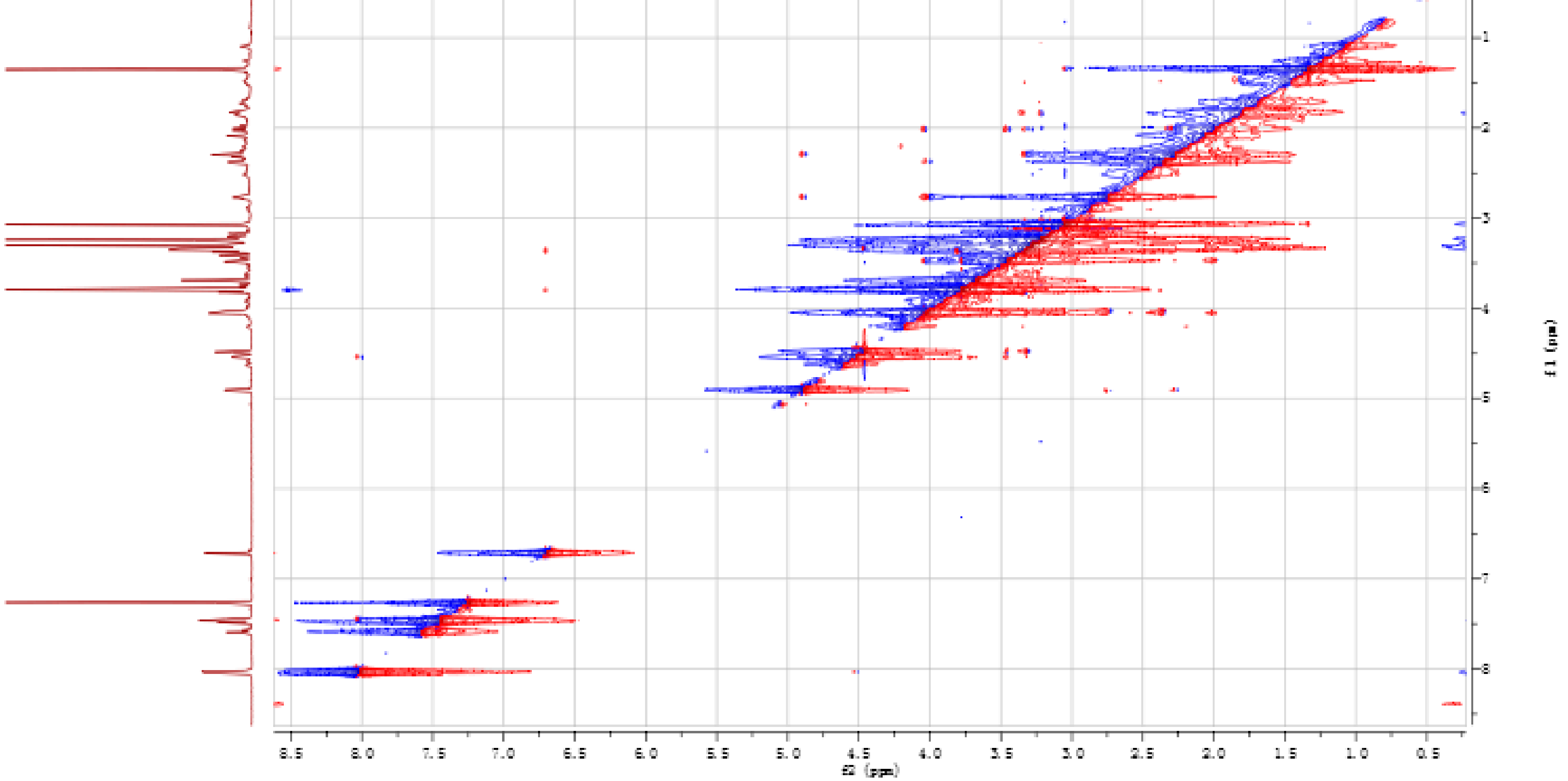

Figure S16. The ROESY spectrum of **2** (400MHz in $CDCl_3$).

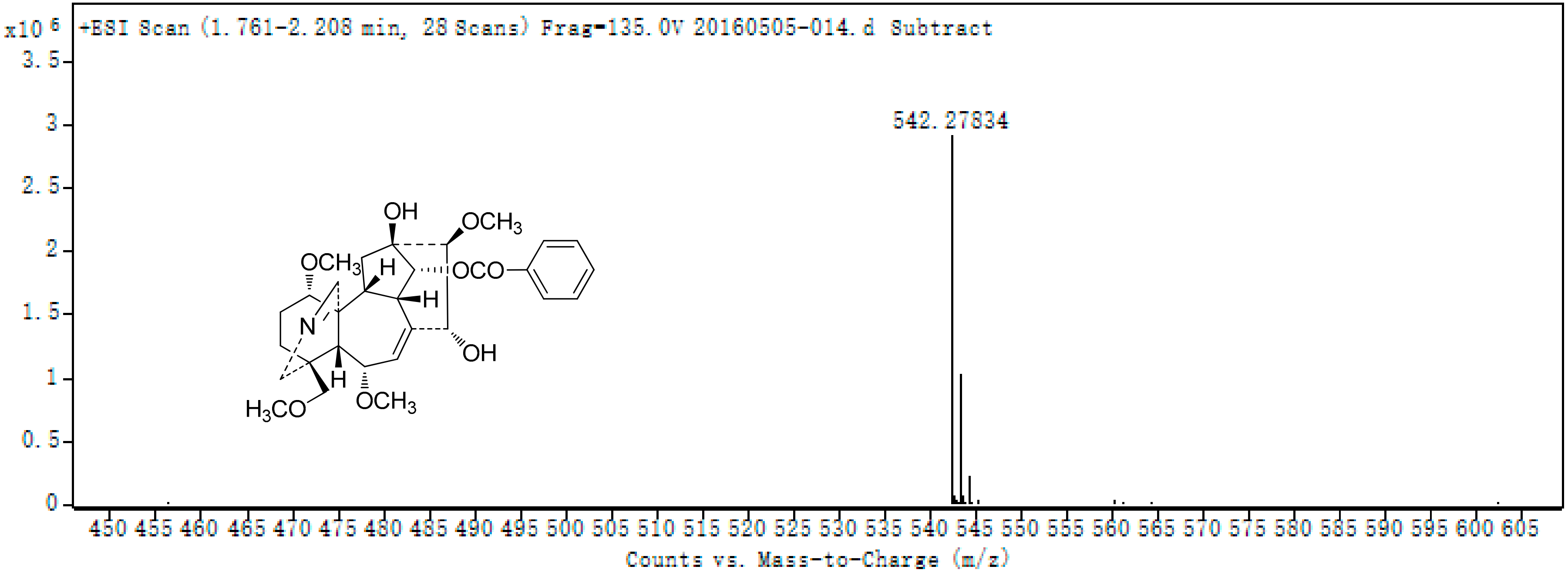

Figure S17. The HR-ESI-MS spectrum of **3** (in MeOH).

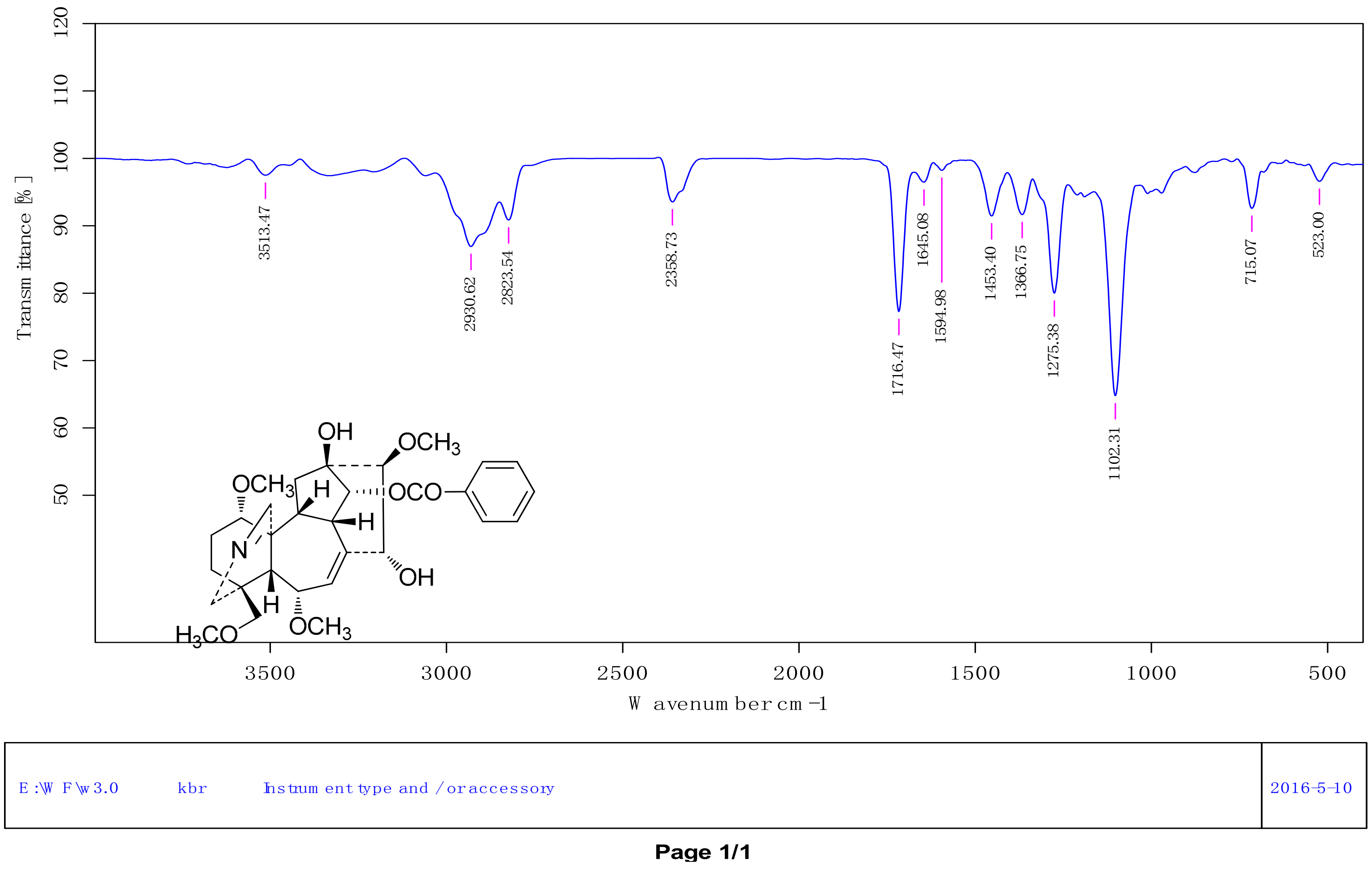

Figure S18. The IR spectrum of **3** (in KBr).

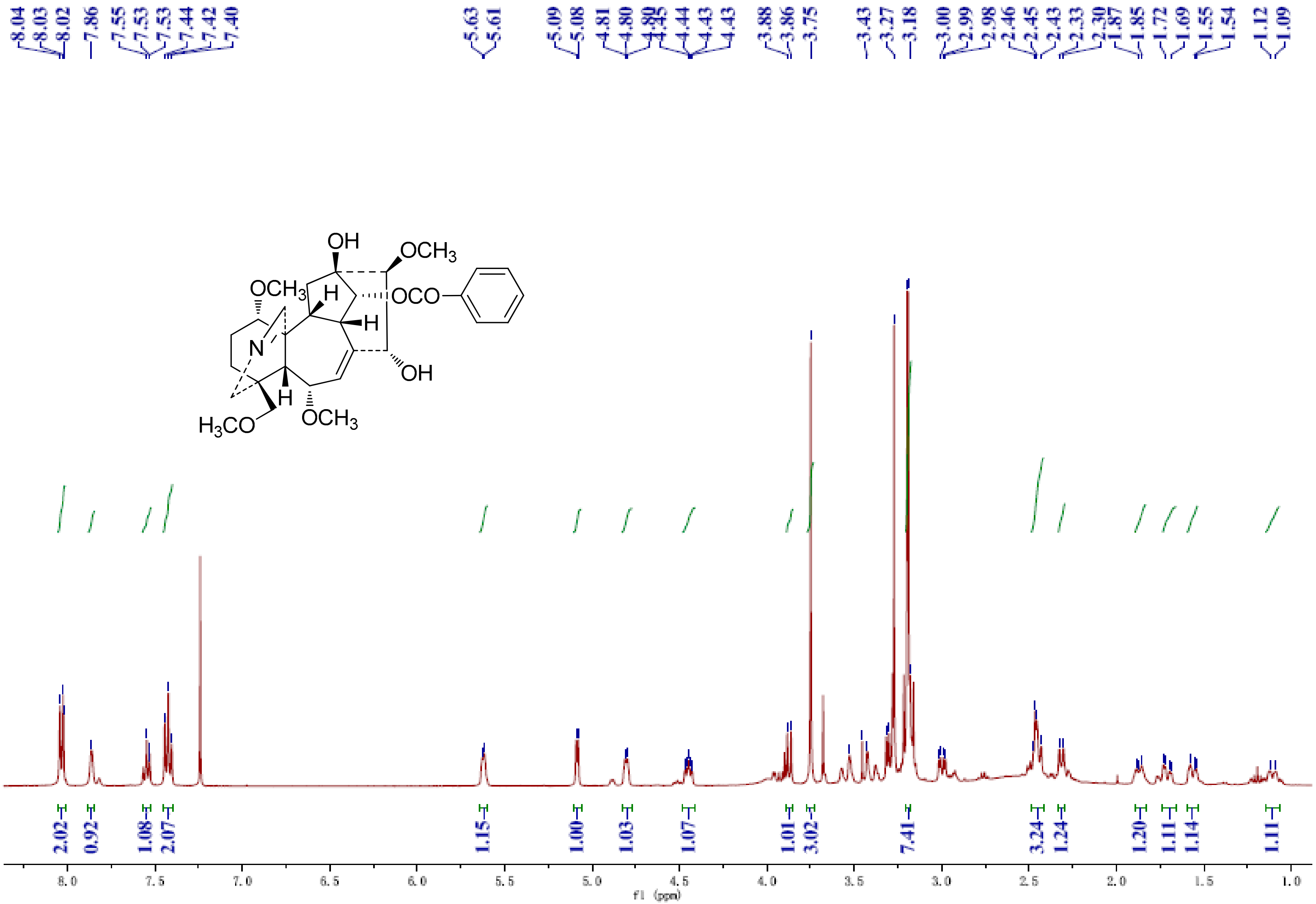

Figure S19. The ^{1}H-NMR spectrum of **3** (400MHz in $CDCl_3$).

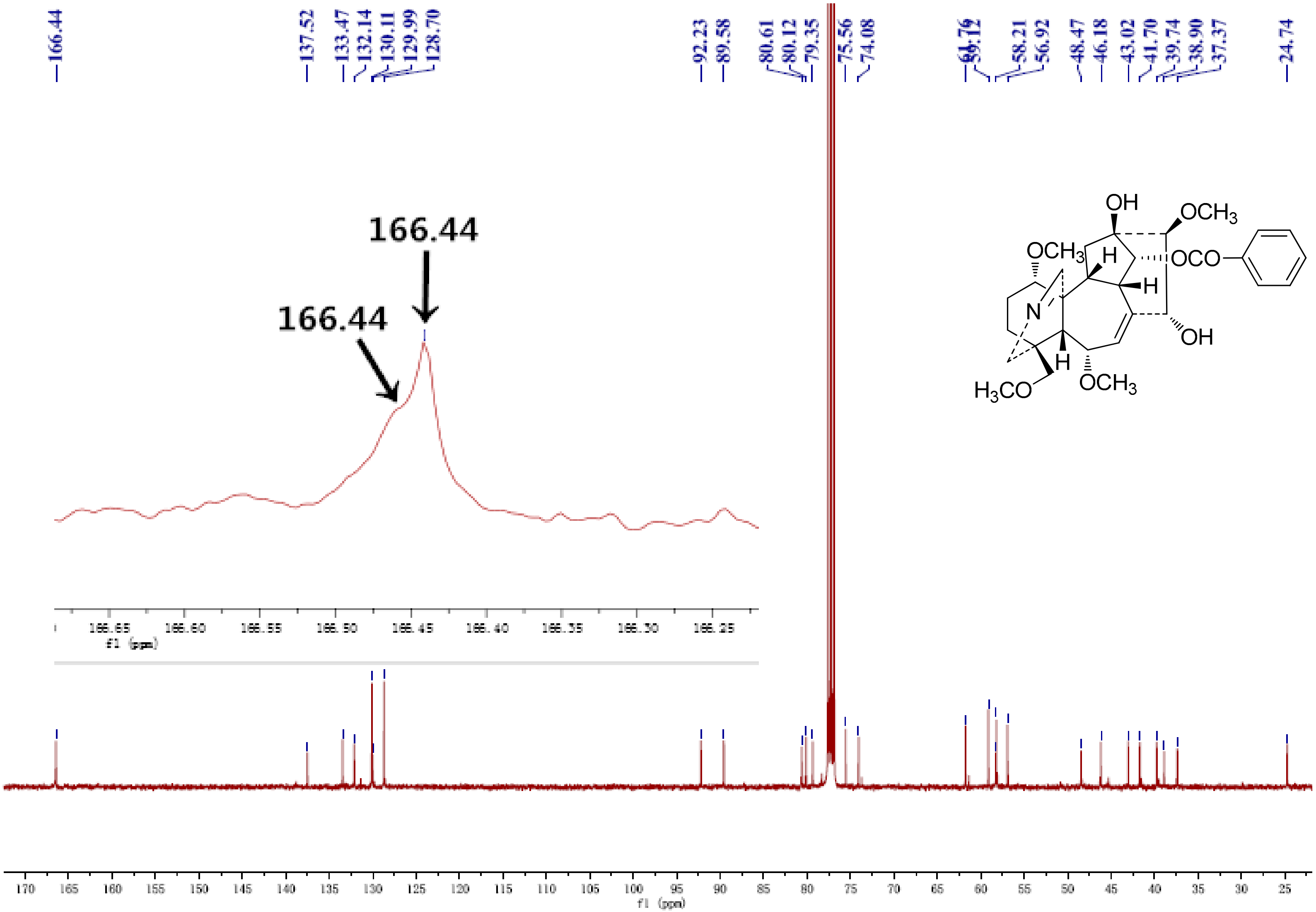

Figure S20. The ^{13}C-NMR spectrum of **3** (100MHz in $CDCl_3$).

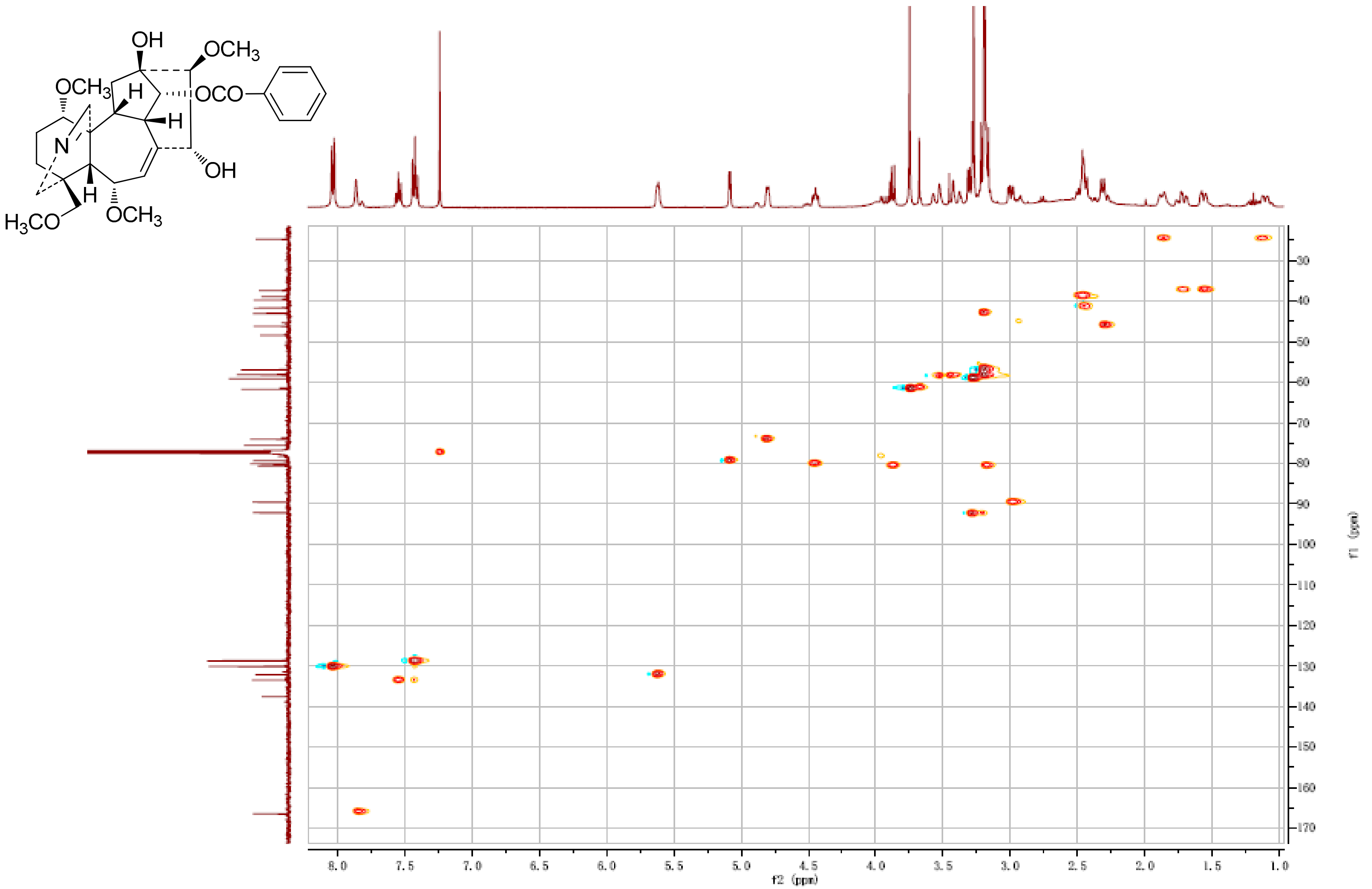

Figure S21. The HSQC spectrum of **3** (400MHz in $CDCl_3$).

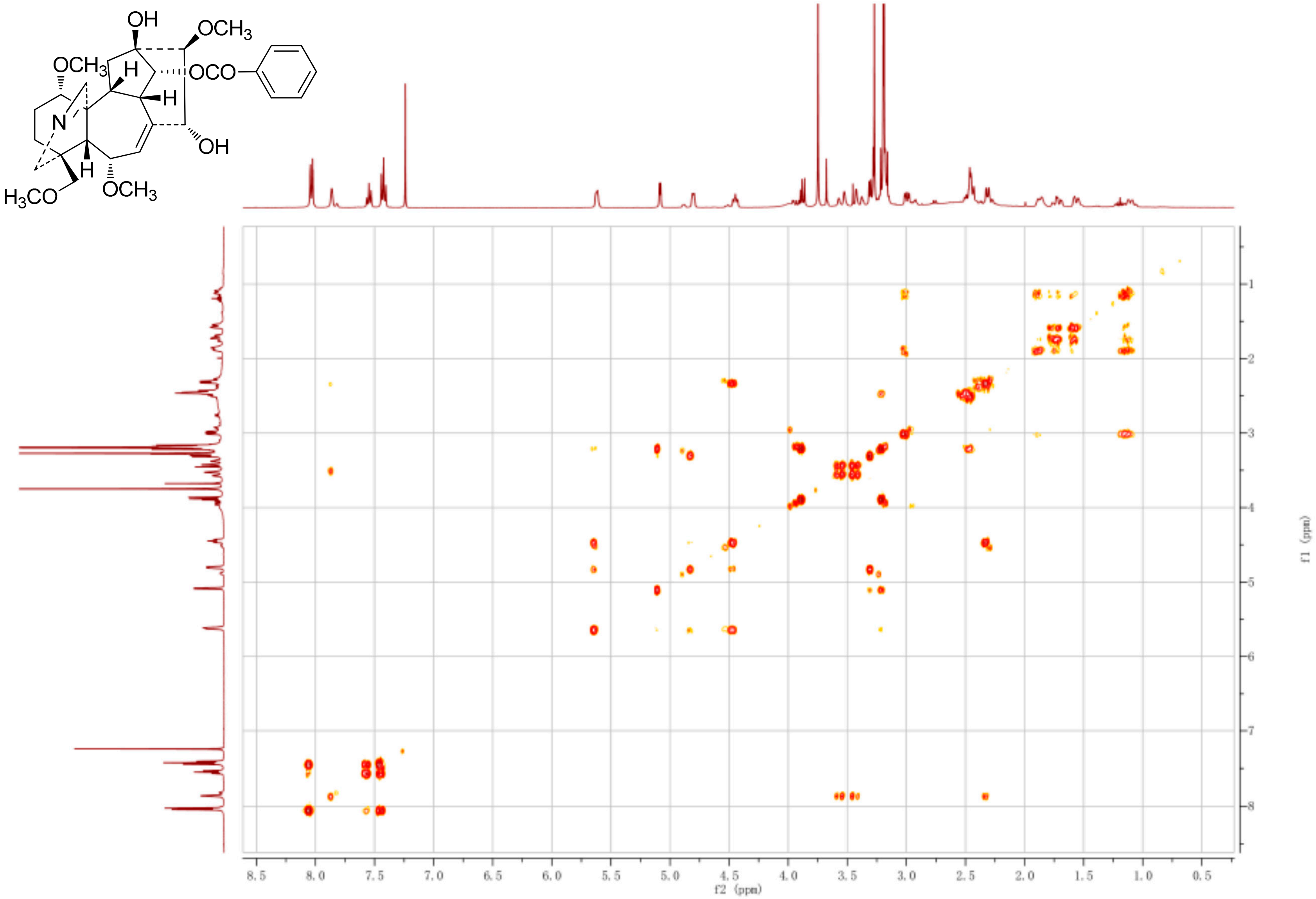

Figure S22. The ^{1}H-^{1}H COSY spectrum of **3** (400MHz in $CDCl_3$).

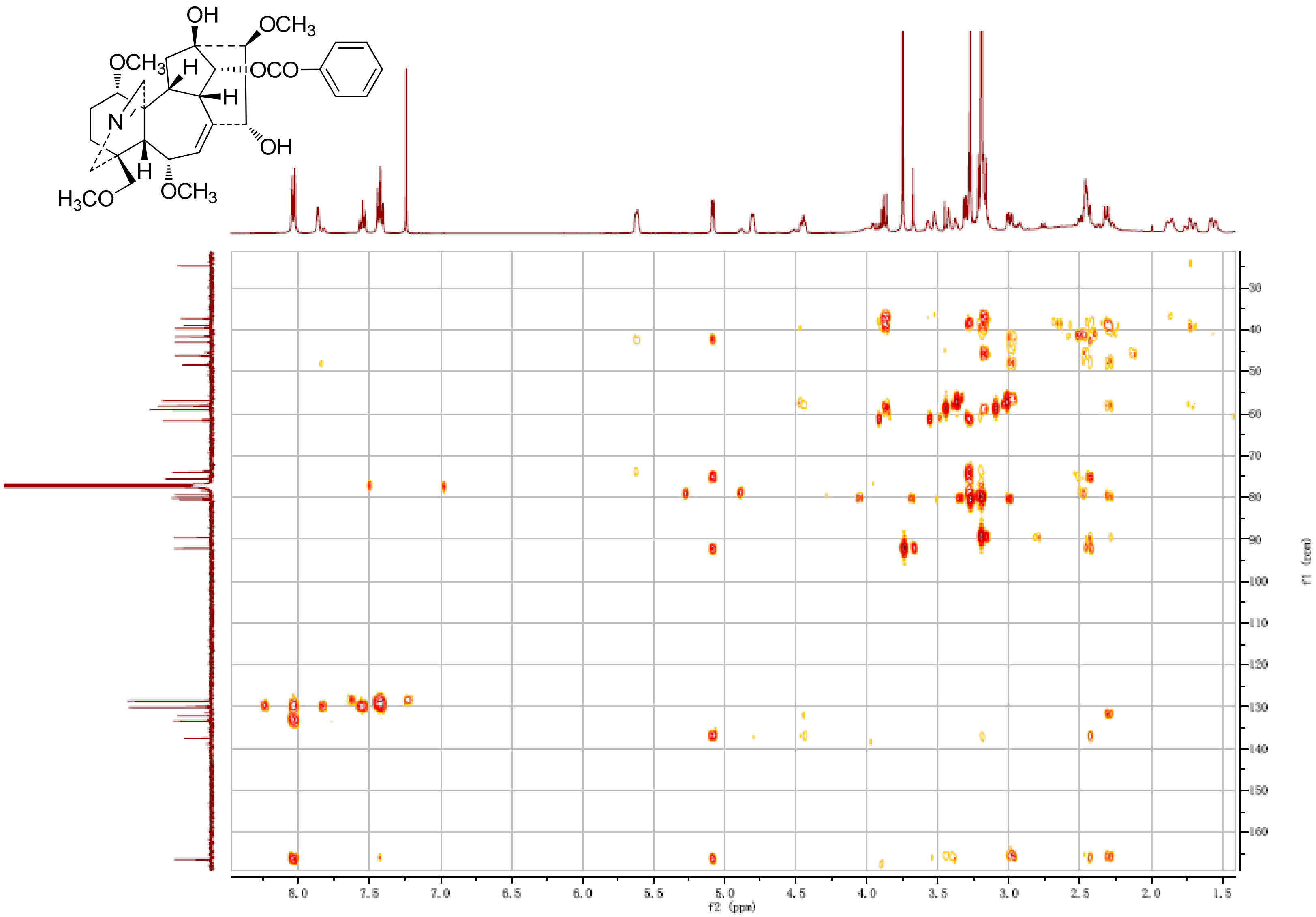

Figure S23. The HMBC spectrum of **3** (400MHz in CDCl3).

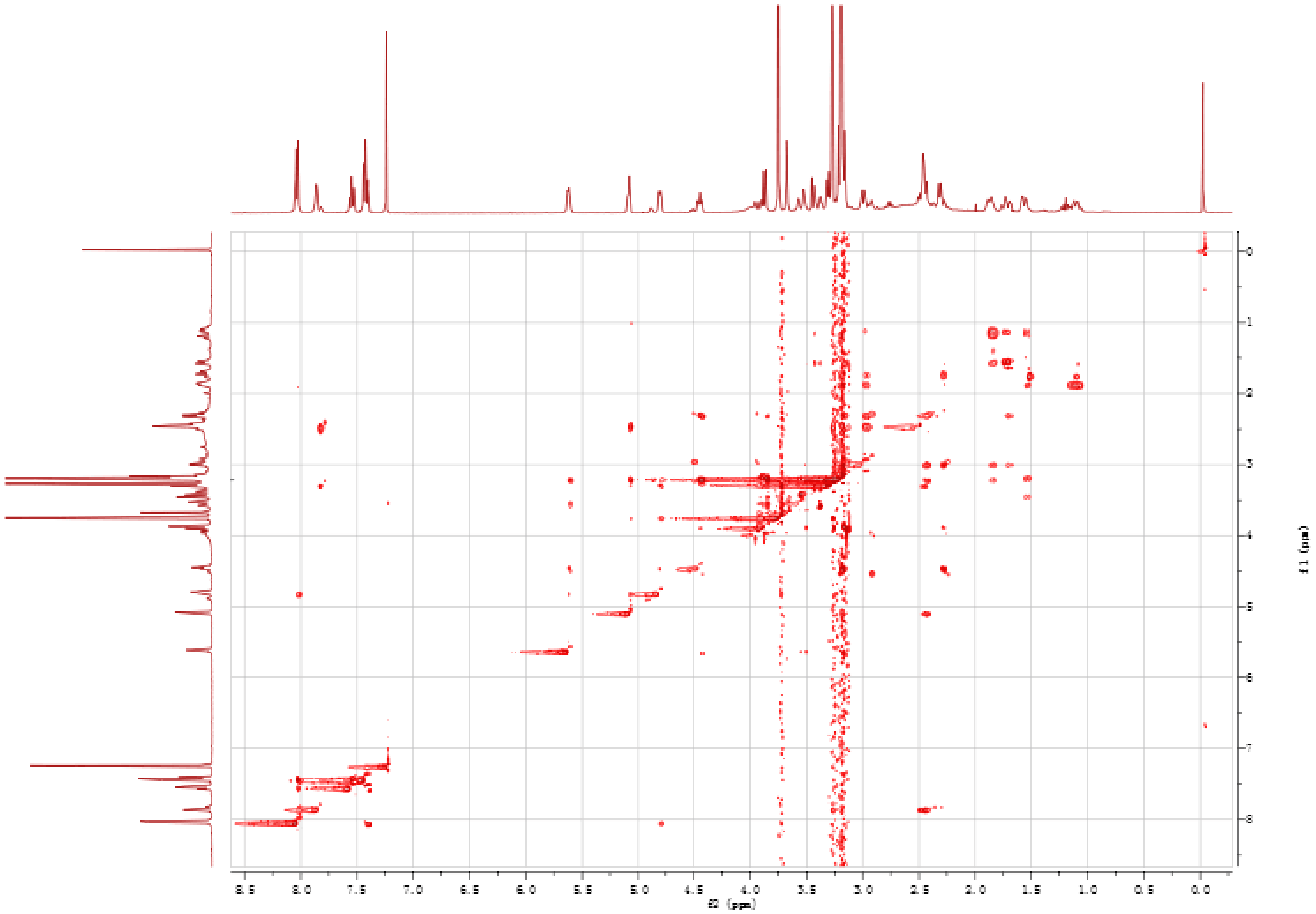

Figure S24. The ROESY spectrum of **3** (400MHz in $CDCl_3$).
